# The accessibility and acceptability of self-management support interventions for men with long term conditions: a systematic review and meta-synthesis of qualitative studies

**DOI:** 10.1186/1471-2458-14-1230

**Published:** 2014-11-27

**Authors:** Paul Galdas, Zoe Darwin, Lisa Kidd, Christian Blickem, Kerri McPherson, Kate Hunt, Peter Bower, Simon Gilbody, Gerry Richardson

**Affiliations:** Department of Health Sciences, University of York, York, YO10 5DD UK; School of Healthcare, University of Leeds, Leeds, LS2 9JT UK; School of Health and Life Sciences/Institute for Applied Health Research, Glasgow Caledonian University, Glasgow, G4 0BA UK; NIHR School for Primary Care Research, Manchester Academic Health Science Centre, University of Manchester, Manchester, UK; MRC/CSO Social and Public Health Sciences Unit, University of Glasgow, Glasgow, G12 8QQ UK; Centre for Health Economics, University of York, York, YO10 5DD UK

**Keywords:** Men’s health, Long term conditions, Self-management, Masculinity

## Abstract

**Background:**

Self-management support interventions can improve health outcomes, but their impact is limited by the numbers of people able or willing to access them. Men’s attendance at existing self-management support services appears suboptimal despite their increased risk of developing many of the most serious long term conditions. The aim of this review was to determine whether current self-management support interventions are acceptable and accessible to men with long term conditions, and explore what may act as facilitators and barriers to access of interventions and support activities.

**Methods:**

A systematic search for qualitative research was undertaken on CINAHL, EMBASE, MEDLINE, PsycINFO and Social Science Citation Index, in July 2013. Reference lists of relevant articles were also examined. Studies that used a qualitative design to explore men’s experiences of, or perceptions towards, self-management support for one or more long term condition were included. Studies which focused on experiences of living with a long term condition without consideration of self-management support were excluded. Thirty-eight studies met the inclusion criteria. A meta-ethnography approach was employed to synthesise the findings.

**Results:**

Four constructs associated with men’s experience of, and perceptions towards, self-management support were identified: 1) need for purpose; 2) trusted environments; 3) value of peers; and 4) becoming an expert. The synthesis showed that men may feel less comfortable participating in self-management support if it is viewed as incongruous with valued aspects of their identity, particularly when activities are perceived to challenge masculine ideals associated with independence, stoicism, and control. Men may find self-management support more attractive when it is perceived as action-oriented, having a clear purpose, and offering personally meaningful information and practical strategies that can be integrated into daily life.

**Conclusions:**

Self-management support is most likely to be successful in engaging men when it is congruent with key aspects of their masculine identity. In order to overcome barriers to access and fully engage with interventions, some men may need self-management support interventions to be delivered in an environment that offers a sense of shared understanding, connectedness, and normality, and involves and/or is facilitated by men with a shared illness experience.

**Electronic supplementary material:**

The online version of this article (doi:10.1186/1471-2458-14-1230) contains supplementary material, which is available to authorized users.

## Background

The care and treatment of people living with a long term condition (LTC) – a condition or disease that cannot currently be cured but can be managed through medication, therapy and/or lifestyle modification, such as diabetes, arthritis and heart failure – is a major worldwide public health concern. In the UK, over 15 million people currently have a LTC [1] and this number is set to increase over the next decade, with significant rises in multi-morbidity [2].

The increasing burden of LTCs is leading to a shift in emphasis in healthcare delivery towards the promotion of self-management as a critical element of LTC care and a key mechanism for ensuring that future service delivery remains effective, efficient and sustainable [2–4]. Self-management refers to an individual’s ability to effectively manage the symptoms, treatment, physical and psychosocial consequences and lifestyle changes associated with living with a LTC [5]. Self-management support (SMS) interventions can be defined as those which focus on developing the abilities of patients to undertake effective self-management through education, training and support to develop knowledge, skills or psychological and social resources [6].

The evidence base on SMS interventions is rapidly expanding and a wide range of interventions have been developed, from skills-based training for specific conditions such as type 1 diabetes (DAFNE [7]) and type 2 diabetes (DESMOND [8]) to assistive technologies such as telehealth and telecare [9]. Other interventions include lay-led support programmes for generic LTCs such as the UK Expert Patient Programme [10] based on the Stanford Chronic Condition programme [11], which aims to promote behavioural change by improving the confidence (self-efficacy) of individuals to manage the physical and psycho-social effects of LTCs. A number of systematic reviews have been carried out on different aspects of SMS. These have focused on interventions targeting specific conditions (e.g. diabetes or mental health) [12, 13] types of intervention (e.g. lay-led programmes) [14], or on particular outcomes (e.g. medicines adherence) [15] and have shown benefits in clinical, lifestyle and psychosocial outcomes. Delivered on a large scale, the evidence suggests that SMS interventions have the potential to reduce healthcare costs, achieve effective redistribution of services from hospital to the community, and optimise health outcomes for people with LTCs [2, 16, 17]. However, despite a developing evidence base on the effectiveness of SMS, major knowledge gaps remain, particularly around patient engagement and what works, for whom, and why [4, 17].

The effectiveness of SMS is considerably limited by the numbers of patients able or willing to access and engage with available interventions [4, 18, 19]. SMS interventions often fail to engage a significant number, or specific sub-populations, of the wider population because they are not personalized to, or grounded within, the contexts and everyday lives of the individuals and settings in which they interact with health professionals and in which self-management decision making occurs [20].

Men, as a group, are frequently underrepresented at many SMS services [10, 14, 21–23] and are believed to be poorer self-managers than women [24–29] despite having an increased incidence of many of the most serious and disabling LTCs such as chronic pulmonary disease, diabetes and cardiovascular diseases [25, 30]. This is consistent with a growing body of research which shows that risky or unhealthy behaviours (e.g. drinking, smoking, reticence to access health services) are closely related to ‘traditional’ masculine attitudes that emphasise self-sufficiency, stoicism and robustness [31, 32] and are associated with men’s poorer health outcomes compared to women [24–29, 33]. Recognition of this trend and the increased incidence of serious LTCs in men have led to widespread calls and urgent action for interventions to be specifically targeted at men, in general [25, 32, 33]. For this reason, we conducted a systematic review and meta-synthesis of the qualitative research literature to examine the experiences of, and perceptions towards, SMS among men with LTCs. We aimed to determine whether current SMS interventions are acceptable and accessible to men with LTCs and explore what may act as facilitators and barriers to access of interventions and support activities. Results from a parallel quantitative review of the effectiveness of SMS interventions in men are reported elsewhere.

## Methods

We undertook a systematic search of qualitative literature and employed a meta-ethnography approach to synthesis based on the methods described by Noblit and Hare [34] and Campbell and colleagues [35]. As this was a secondary synthesis of data, ethical approvals were not required.

### Search strategy

A comprehensive electronic search strategy was developed in liaison with information specialists that sought to identify all relevant studies. Five electronic databases were searched in July 2013 (CINAHL, EMBASE, MEDLINE, PsycINFO and Social Science Citation Index).

Due to challenges with methodological indexing of qualitative research [36], the electronic search was complemented by checking reference lists of pertinent papers, and used an adaption of a strategy published elsewhere [37] that included ‘thesaurus terms’ (keywords indexed in electronic databases, e.g. “Qualitative Research”), ‘free text terms’ (commonly used research methodology terms searched for in the titles, abstracts, keywords) and ‘broad-based terms’ (i.e. the broad free-text terms “qualitative”, “findings”, “interview$” and the thesaurus term “Interviews”). Terms relating to gender were combined with other terms to increase the precision of the strategy (e.g. “men”, “male”, “masculin$”, “gender”, “sex difference$”, “sex factors”).

### Study screening and inclusion criteria

Records were initially screened against the broad inclusion criteria by one reviewer (ZD) on the basis of the title and abstract. All articles identified as potentially eligible for inclusion were obtained in full. Attempts were made to identify and obtain published findings for unpublished literature that was otherwise eligible; for example, PhD theses or conference proceedings. The full text literature was screened independently by two reviewers (ZD and PG) to identify studies that met all of the following inclusion criteria:

- Presented analysis of qualitative data
- Written in English and published and peer-reviewed in an academic journal
- Participants identified as having one or more LTC
- Data collected in relation to SMS activities and interventions
- Sample either male only or mixed gender (with explicit comparison by gender)
- Sample comprised of adults (or predominately adults)

Studies that used mixed gender samples but did not offer a clear and explicit comparison between men and women were excluded. Also excluded were studies which focused on self-management experiences of people with LTCs more generally (e.g. those which examined ‘lived experience’ without consideration of SMS).

### Quality appraisal and data extraction

The purpose of quality appraisal in the review was to assess the strengths and weaknesses of the included studies rather than as a basis for inclusion/exclusion. We took the stance of Campbell and colleagues [35] that studies of weaker quality either would not contribute, or would contribute only minimally, to the final synthesis. With that in mind, we used the Critical Appraisal Skills Programme (CASP) tool [38] to assess the quality of various domains (including aims, design, methods, data analysis, interpretation, findings and value of the research). Some additional questions, informed by other meta-ethnography studies [35, 39], were used as prompts to facilitate summaries of the main strengths and limitations of each study. Appraisal was conducted by two reviewers independently (ZD and PG) with disagreements resolved through discussion.

### Data extraction and synthesis

All study details (including aim, participant details, methodology, method of data collection, and analysis) were initially extracted by one reviewer (ZD) using data extraction forms previously tested and refined through discussion within the review team following a pilot study of four papers. All data extraction forms were double-checked for accuracy by a second reviewer (PG). We used a meta-ethnography approach to synthesis that broadly followed the steps described by Noblit and Hare [34]. The analytical process involved three levels of ‘construct’ [39, 40]:

i. First-order constructs: Participant quotes and participant observations, whilst recognising that in secondary analysis these represent the participants’ views as selected by the study authors in evidencing their second-order constructs.
ii. Second-order constructs: Study authors’ themes/concepts and interpretations, also described by Noblit and Hare as ‘metaphors’.
iii. Third-order constructs: the review team’s new interpretations of original authors’ interpretations, based on our analysis of first-order and second-order constructs extracted from the studies.

First-order and second-order constructs from each paper were initially imported into *NVivo10* and grouped into broad categories of SMS intervention/activity by one reviewer (ZD) to offer a ‘way in’ to the synthesis [35]. Each ‘group’ of studies were then coded inductively by pairs of reviewers, each of whom independently completed matrices to report the second-order constructs and any emerging third-order constructs for each paper. Peer debriefing meetings were then held between reviewers to discuss coding, facilitate the consideration of alternative interpretations, and agree on the second- and third-order constructs, which were subsequently imported into *NVivo*. Original authors’ words were retained in second-order constructs wherever possible. The final third-order constructs were developed during a full-day meeting of the entire review team, where the coded constructs were systematically compared and translated into one another (see Additional file 1). The process involved several iterations until a ‘line of argument’ synthesis, which took into account the similarities and differences evident in the studies, was agreed. Finally, the synthesis was refined in discussions with our Patient and Public Involvement (PPI) group which comprised five men with LTCs who had provided guidance and input throughout the review process.

## Results

### Study characteristics

**Figure 1:**
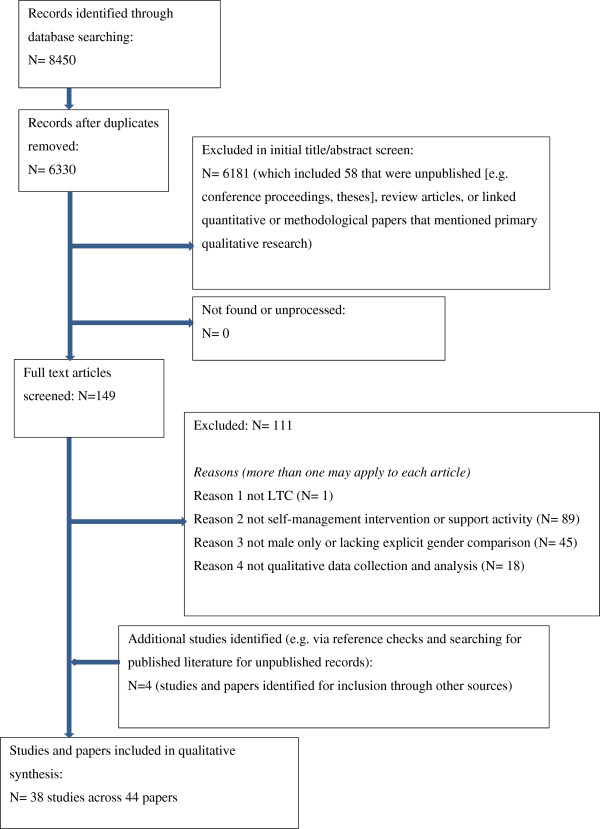
**PRISMA flow diagram for systematic literature search.**

The electronic search strategy identified 6330 unique references. Screening based on title/abstracts identified 149 articles for full text screening. Dual screening of these full text articles identified 34 studies (reported in 38 articles) that were included in the review. An additional four studies were identified through the checking of reference lists, giving a total of 38 studies (reported in 44 articles) included in the final review (see Figure 1).

Twenty-six of the studies comprised male-only samples; the other 12 studies comprised mixed-sex samples that included explicit comparison between men and women. The majority were conducted in the USA (n = 13 studies) and the UK (n = 11), with the remainder in Australia (n = 5), Canada (n = 5), and one in each in Denmark, France, South Africa, and Sweden.

The most common LTC considered in the studies was cancer (n = 22), followed by HIV/AIDS (n = 7), cardiac conditions (n = 4: coronary artery disease n = 1, heart failure n = 1, myocardial infarction n = 2), mental health (n = 2: depression n = 1, depression/anxiety n = 1), arthritis (n = 1), type 2 diabetes (n = 1) and multiple sclerosis (n = 1).

The most common type of SMS interventions were face-to-face support groups (12 studies), followed by ‘lifestyle’ interventions (11 studies) and internet information and/or online support (5 studies). The remaining studies concerned any experiences of ‘any’ SMS, including experiences of psychosocial support services, one-to-one support, and peer support, as well as views on potential interventions of perceived benefit (see Tables 1 and 2).

**Table 1:** **Categories and descriptions of self-management interventions and support activities**

Self-management category	Description
**Face-to-face support group**	Any face-to-face support group. This could include peer or professional-led groups and groups that were time-limited or rolling in nature. These groups usually involved sharing of personal information and experiences, sometimes including lectures or question-answer sessions. Groups classified here did not include activities such as physical activity or practising stress management techniques.
**Internet information and/or support**	Any Internet-based support activity, involving support through forums and discussion boards and/or information, either through boards or searching websites.
**Information (including online)**	Any use of information, regardless of source.
**Psychological**	Any intervention or activity with a clear psychological component (e.g. professional counselling) and/or described by the authors as psychological.
**Lifestyle**	Any intervention or activity that includes components of training and/or education which seek to address behaviour change (e.g. physical activity, diet, medication-taking).
**Various**	Any combination of activities (e.g. any self-management services; counselling and peer support).

**Table 2:** **Characteristics of included studies**

Study (First Author, Year, Country)	Aim	Classification of support activity used in qualitative synthesis	Condition	Data collection (IV, FG, OP, PO) and sample size	Methodological approach	Sample (size, sex, condition details, age, ethnicity, locality/settlement, SES, employment, sexuality, relationship)
**Adamsen** [41] **Denmark**	Men's experiences of a tailored intervention involving physical activity and information relay for men with cancer	lifestyle	cancer - any	FG 10 men, PO 17 men, Total 17 men	descriptive/interpretive	range of cancers and varying stages; mean age 56.5 yrs (range 21-71); ethnicity n/r; “broad range” of education, employment, relationship status
**Arrington** [42] **USA**	Communication practices of Man to Man prostate cancer support groups	support group (face-to-face)	cancer - prostate	PO n/r (20 groups of men)	discourse analysis	all “senior citizens”; “almost exclusively retired, elderly”; no further details reported
**Baird** [43] **USA**	Self-care factors influencing adherence to a cardiac rehabilitation programme	lifestyle	cardiac - coronary artery disease	IV 5 men	phenomenology	majority myocardial infarction (4 myocardial infarction, 1 sudden cardiac arrest; one was Post-Cerebral-Vascular Accident (CVA); two were post-percutaneous-transluminal-coronary angioplasty, and one was post-coronary-artery-bypass-graft surgery); aged 60-70 yrs; 80% Caucasian (4 Caucasian, 1 African American); 100% retired; mix of previous 'blue-collar' and 'white-collar' occupations; 100% married
**Barlow** [44] **UK**	Patients' with multiple sclerosis experiences of the Chronic Disease Self-Management Course	lifestyle	multiple sclerosis	IV 3 men 7 women	descriptive/interpretive (part of mixed methods study)	disease duration 4-19 yrs; aged 35-60 yrs
**Barlow** [45] **UK**	Patients' with myocardial infarction experiences of the Chronic Disease Self-Management Course and cardiac rehabilitation	lifestyle	cardiac - myocardial infarction	IV 10 men 9 women	descriptive/interpretive (part of mixed methods study)	14 with co-morbidity; median age 68 yrs (range 59-74); 1 employed; majority (16) married/ residing with partner; all had attended at least 5 of the 6 intervention sessions.
**Bedell** [46] **USA**	Daily life experiences of gay men with HIV/AIDS living alone in New York City	support group (face-to-face)	HIV/AIDS	IV 8 men	descriptive/interpretive	varying severity (6 diagnosed with AIDS for ≥ 2 yrs, 2 not yet developed); all had prior/current difficulty with daily activities; aged 25-50 yrs; majority white (6 white, 2 African American); all urban; majority “middle-class”; education ranged 1 yr college to doctorate; 4 employed, 3 on public assistance, 1 neither; all gay; all lived alone.
**Bell** [47] **Canada**	Composition, processes and patients' views of differently designed and structured cancer support groups	support group (face-to-face)	cancer – 1) women with metastatic cancer; 2) Colorectal cancer patients; 3) Chinese patients with cancer)	IV 3 men and 17 women. PO Metastatic group: 0 men, 25 women; Colorectal group: 14 men, 16 women; Chinese group: 35 men, 61 women (incl. 48 caregivers).	descriptive/interpretive	interview sample: time since diagnosis 3 months-3 yrs, 13 in treatment, 7 post treatment; time in group 1 month-4yrs; median age 50s (range 40s-70s); ethnicity n/r; metastatic observation sample: 0 men, 25 women; 25 in treatment; median age 50s (range 30s-60s); majority white; colorectal observation sample: 14 men, 16 women; 1 pretreatment, 8 in treatment, 12 post treatment; median age 50s (30s-70s); majority white; Chinese observation sample: 35 men; 61 women; 5 pretreatment, 30 in treatment, 15 post treatment; median age 50s (20s-80s); all Chinese.
**Bourke** [48] **UK**	Men's experiences of a lifestyle intervention for men with prostate cancer undergoing androgen suppression therapy	lifestyle	cancer - prostate	FG 12 men (3 groups)	descriptive / interpretive	all T3-T4 prostate cancer receiving androgen suppression therapy ≥ 6 months; details n/r but linked trial reports for intervention group of 25: mean treatment 30 months (sd 31); mean age 71.3 yrs (sd 6.4)
**Broom** [49] **Australia**	Impact of Internet use on disease experience of prostate cancer and the doctor-patient relationship	Internet (information and/or support)	cancer - prostate	I V33 men	descriptive / interpretive	“range” of prognoses and treatments; “varying ages”
**Chambers** [50] **Australia**	Men's experiences of a mindfulness-based cognitive therapy group intervention in men with advanced prostate cancer	psychological	cancer - prostate	IV 12 men	descriptive / interpretive	n/r for interview sample therefore based on 19 men taking part in intervention. Time since diagnosis mean 68.9 months (sd 51.2, range 1-167); majority had hormone treatment; range of surgery and radiotherapy (16 received hormone therapy incl. 9 ongoing; 11 external beam radiation therapy, 3 brachytherapy, 3 radical prostatectomy surgery, 1 orchidectomy); mean age 67.0 yrs (sd 6.5 yrs, range 58-83); 79% completed university, college, or vocational training; 37% employed, 63% retired; 84% married or in a relationship, 16% widowed, divorced, or separated.
**Chenard** [51] **USA**	Impact of stigma on self-care behaviours of HIV-positive gay men	support group (face-to-face)	HIV/AIDS	IV 15 men, FG 5 men (1 group), Total 20 men	grounded theory	all HIV+ ≥1 yr, 85% ≥5 yrs; median age 44 yrs (range 26-62; 70% over 30 yrs); all gay.
**Corboy** [52] **Australia**	Perceived barriers to using psychosocial support services in men with cancer living in rural Australia	various	cancer - prostate	IV 9 men (82 surveyed and subsample interviewed)	descriptive / interpretive	men with ‘any’ cancer eligible but all participants had prostate cancer; mean age 69 yrs (sd 9.3); all rural (5 accessible, 4 moderately based on ARIA+ classification); all married; 2 employed, 1 sick leave, 6 retired.
**Cramer** [53] **UK**	Men's experiences of depression and anxiety groups and the role of health professionals in accessing support	support group (face-to-face)	depression/anxiety	IV 17 men, PO 30 (4 groups, unclear if this includes some women), Total 38 (may include women)	descriptive / interpretive	details n/r; sampling described as aiming to increase ethnic diversity and diversity in type of help sought
**Dickerson 2006** [54] **USA (linked study to Dickerson** [55] **)**	Experiences of patients with cancer using the Internet for information and support to manage self-care, including symptom management	Internet (information and/or support)	cancer - any	IV 20 women (intended as mixed but only managed to recruit women) - linked study	phenomenology	various cancer types (11 breast, 3 gynecologic, 1 gastrointestinal, 3 lymphomas, 2 hematological; 7 new diagnosis, 7 in treatment, 6 survivors (>5 yrs); mean age 52.3 yrs (sd 8.7, range 34-65); mean education 15 yrs (sd 2, range 12-18); mean 14 hours weekly Internet use (sd 12, range 2-40); mean 6 yrs using Internet (range 2-10).
**Dickerson** [55] **USA**	Experiences of men with cancer using the Internet	Internet (information and/or support)	cancer - any	IV 15 men (comparison made with 20 women in above study)	phenomenology	majority prostate cancer (14 prostate, 1 leukemia); 1 new diagnosis, 4 in treatment, 10 survivors (>5 yrs); mean age 63 yrs (sd 10, range 47-78); mean education 17 yrs (sd 3, range 12-20); mean 11 hours weekly Internet use (sd 10, range 1-35); mean 7 yrs using Internet (range 1-10); 10 attend 'Us, Too' face-to-face support group
**Eldh** [56] **Sweden**	Phenomena of participation and non-participation in nurse-led clinic for chronic heart failure, as observed in visits and experienced by patients and nurses	lifestyle	cardiac - heart failure	IV 3 men, PO 3 men (11 visits), Total 3 men	phenomenology	classed as II/III using New York Heart Association classes of heart failure; aged 53, 77, 79 yrs
**Emslie** [57] **UK**	Experiences of men and women with depression in articulating emotional distress and engaging with health professionals	various	depression	IV 16 men 22 women	descriptive/interpretive	majority (34/38) experienced multiple/prolonged depressive episodes; 18/38 hospitalised; 10/38 bipolar depression; wide age range (<30-66+; 3 <30, 14 30-40, 11 41-55, 6 56-65, 4 66+ yrs); majority White British (33/38 - others 1 each of Black, Asian, South European, North European, American).
**Evans** [58] **UK**	Acquisition and evaluation of complementary and alternative medicine (CAM) information in men with cancer	information	cancer - any	IV 34 men (total sample is 43 but paper focus is on 34 who did use CAM)	descriptive/interpretive	various cancer types (10 colorectal, 10 prostate, 3 lung, 11 other - thymic, tonsillar, pancreatic, bone, lymphoma, bladder, renal, oesophageal, leukaemia); varying stages (10 localised, 10 remission, 8 metastatic, 6 palliative care); mean age 57 yrs (range 31-83), all white; 'range' of manual, non-manual and professional occupational backgrounds (over half professional); 22 used CAM before diagnosis.
**Ferrand** [59] **France**	Motives for regular physical activity in men and women with type 2 diabetes using the French patients' association Move for Health	lifestyle	diabetes - type 2	IV 9 men 14 women	descriptive/interpretive	men: 6 diagnosed ≥5 yrs; 6 medicated including 2 insulin; mean age 67.0 yrs (sd 6.1); 6 post-secondary education; 1 employed, 8 retired; 7 married, 1 widowed, 1 never married; women mean 56.3 yrs (sd 9.5), total range 35-78 yrs; 13 diagnosed ≥5 yrs; 10 medicated including 4 insulin; mean age 56.3 (sd 9.5); 4 post-secondary education; 3 employed, 7 retired, 4 homemaker; 7 married, 1 widowed, 5 separated/divorced, 1 never married.
**Galdas** [60] **Canada**	Canadian Punjabi Sikh men's experiences of adopting lifestyle changes following myocardial infarction	lifestyle	cardiac - myocardial infarction	IV 27 men	descriptive/interpretive	majority reported comorbidity (10 diabetes, 8 high blood pressure, 7 high cholesterol, 3 depression); mean age 65.7 yrs (range 41-86); all Canadian Punjabi Sikh; lived in Canada mean 20 yrs (range 2-42); majority retired, 7 employed, 13 receiving pension or disability income; 24 married, 3 widowed; 15 attended cardiac rehabilitation.
**Gibbs** [61] **Australia**	Factors influencing utilisation of self-management services in men with arthritis	various	arthritis	IV 17 men	grounded theory (and participatory research)	time since diagnosis 4 months-25 yrs; varied health status (self-reported 3 poor, 8 fair, 6 good); median age 41-60 yrs (2 18-25, 3 26-40, 7 41-60, 3 61-75, 2 75+); majority Anglo/Celtic (12 Anglo/Celtic incl. 1 also Aboriginal; 1 UK/European, 1 Greek, 1 Chilean, 1 Italian, 1 Filipino/Asian); varied education (1 primary school only, 5 completed secondary, 1 passed secondary, 7 vocational, 3 university, 3 n/r); 9 employed, 6 retired, 1 student, 1 unemployed; range of employment roles (health services, research, managerial, information technology, motor mechanics, farming); sexuality not asked but 1 homosexual, 1 bisexual, others referred to female partners although acknowledge may not identify as heterosexual; varied involvement in self-management programmes (0 to 4 different programmes).
**Gibbs** [23] **Australia**	Work as a barrier to accessing self-management services in men with a chronic illness (arthritis)	various	arthritis	IV 17 men	grounded theory (and participatory research)	see 2005 paper (pooled)
**Gooden** [62] **Australia**	Comparison of ways in which men with prostate cancer and women with breast cancer share issues online	Internet (information and/or support)	cancer - prostate (men) and breast (women)	OP 77 men (591 postings) 69 women (272 postings)	descriptive/interpretive (part of mixed methods)	no sample characteristics due to methods; however quality of writing in postings suggested “reasonably well educated and articulate”
**Gray** [63] **Canada**	Comparison of men's experiences of prostate cancer self-help groups and women's experiences of breast cancer self-help groups	support group (face-to-face)	cancer - prostate (men) and breast (women)	I V12 men, IV/FG 27 women	descriptive/interpretive	men: “representation from among long-term survivors and men with advanced disease”; aged 45-80; women: range of time since diagnosis (4 <1 year, 11 < 3yrs, 10 longer term); range of severity including 6 with recurrence; aged 33-73 yrs (15 aged <50); all white reflecting groups; “predominantly middle class and well educated” (3 had less that high school).
**Gray** [64] **Canada (linked study to Gray** [63] **)**	Men's experiences of prostate cancer self-help groups	support group (face-to-face)	cancer - prostate	IV 12 men	descriptive/interpretive	see above
**Gray** [65] **Canada (linked study to Gray** [63] **)**	Women's experiences of breast cancer self-help groups	support group (face-to-face)	cancer - breast (women)	IV/FG 27 women - linked study	descriptive/interpretive	see above
**Harris** [66] **Canada**	Experiences of counselling and peer support services in gay men with HIV/AIDS	various	HIV/AIDS	IV 12 men	phenomenology	mean 9.75 yrs since diagnosis (range 4-15); mean age 43 yrs (range 27-56); range of education (4 some high school credits, 5 completed high school, 3 “completed some” university/college education); 7 employed, 2 retired, 3 not working; varied income (5 <$20,000, 3 $30,000-$49,999, 1>$50,000, 3 n/r); all gay (5 previously married to women); all involved in local community-based agencies; most reported following their antiretroviral medications; 6 men had used peer support 1-2 times per week for 8 yrs on average; 7 men had received counselling 1-2 times per fortnight for 4 yrs on average.
**Iredale** [67] **UK**	Perceptions of information needs in men with breast cancer	information	cancer - breast (male)	IV 30 men (subsample of n161 men surveyed in full study)	descriptive only (supplement to quantitative study)	details for interview sample n/r; details for full sample surveyed (n161): mean 35 months since diagnosis (range 2-120); 55% current breast cancer; mean age 67.3 yrs (range 27-88); 64% secondary education or above; 78% married/ residing with partner, 8% single, 6% divorced/separated, 8% widowed.
**Kendall** [68] **USA**	Experiences of community support groups in gay men with HIV/AIDS	support group (face-to-face)	HIV/AIDS	IV 29 men	descriptive/interpretive	mean 3 yrs 2 months since HIV diagnosis (range 3 months-9 yrs); range of disease severity (8 asymptomatic; 8 mild, transient symptoms; 8 “full-blown AIDS, not terminal”, 8 “full-blown AIDS in terminal stage”); mean age 37 yrs (range 25-58); majority Caucasian (27 Caucasian, 2 African American); majority highly educated (mean 16 yrs education; only 1 without college education); 53% employed, 46% disability allowance; all gay; 11 in a relationship (length ranging 1 month-14 yrs); 31% strong family support, 46% strong friend support but “in general … did not feel well-supported”; mean 3 HIV-support groups attended (range 1-8).
**Kronenwetter** [69] **USA**	Men's experiences of a prostate cancer lifestyle trial for men with early prostate cancer	lifestyle	cancer - prostate	IV 26 men	descriptive/interpretive	mean age 67 yrs (range 50-85); majority Caucasian (>90%); majority college education, university education or “specialised training” (>90%); “over half” retired; 21 (81%) had “partners/spouses”.
**Martin** [70] **UK**	Men's experiences of a nurse-led workshop for men with testicular cancer	lifestyle	cancer - testicular	IV 6 men	descriptive/interpretive	mean age 35 yrs (range 29-45)
**Mfecane** [71] **South Africa**	Phenomenon of therapeutic citizenship in HIV/AIDS support groups, as observed in visits and experienced by men in rural South Africa	lifestyle	HIV/AIDS	IV 25 men; PO n n/r (14 months, presumably includes some women)	ethnography	interview sample: aged 28-50 yrs; all South African; all rural; all “poor, working-class community”; majority unemployed; approximately half single (6 married, 4 cohabitating, 3 partners living apart, 12 single); all had ≥1 child but minority lived with their children.
**Oliffe** [72] **Canada**	Factors influencing sustainability of prostate cancer support groups	support group (face-to-face)	cancer - prostate	PO 333 men (15 groups of men and partners)	ethnography	details of group members n/r
**Oliffe** [72] **Canada**	How prostate cancer support groups simultaneously facilitate health promotion and illness demotion	support group (face-to-face)	cancer - prostate	PO 333 men (15 groups of men and partners), IV 52 men, Total 333 men	ethnography	interview sample: mean 6.8 yrs since diagnosis; majority received treatment (49); mean age 70 yrs (range 53-87); 25 Anglo-Canadian, 25 Northern European; majority retired (42); all attended ≥2 meetings in past year (inclusion criteria); mean 5.3 yrs accessed support groups; 16 were long-term members (had been attending for more than 12 months), and 16 were short-term members (had been attending for less than 12 months); 20 held leadership roles (e.g. facilitator, secretary).
**Oliffe** [73] **Canada**	How men who attend prostate cancer support groups engage with health literacy and consumerism	support group (face-to-face)	cancer - prostate	PO n n/r (16 groups of men and partners), IV 54 men, Total n n/r	ethnography	interview sample: majority received treatment (50); mean age 71 yrs (range 53-87); all Canadian, “many” Northern European ancestry; majority retired (44); all attended ≥2 meetings in past year (inclusion criteria); 16 were long-term members (had been attending for more than 12 months), and 16 were short-term members (had been attending for less than 12 months), 22 held leadership roles (e.g. facilitator, secretary).
**Ramachandra [** [74] **UK**	Acceptability of a brief self-led psychological intervention in patients with cancer	psychological	cancer - metastatic prostate (men) and metastatic breast (women)	IV unclear if 4 men 3 women or 3 men 2 women (46 in total trial - 24 men 22 women; unclear if feedback at 6 weeks or 12 weeks)	descriptive only (supplement to quantitative study)	interview sample details n/r; full trial details: men: mean age 72.4 yrs; women: mean age 60.8 yrs.
**Sandstrom** [75] **USA**	Utilisation of peer support groups by gay men with HIV/AIDS	support group (face-to-face)	HIV/AIDS	IV 25 men	grounded theory	all advanced diagnoses; 10 symptomatic HIV (including 3 with severe complications), 15 diagnosed with AIDS; age ranged 20-56 yrs (7 20s, 11 30s, 6 40s, 1 50s); majority white (2 African American); 12 “attended college or completed college degrees”; 20 urban; all gay; 16 had used support groups at some time, including 9 briefly (“usually 1-4 months”) and 7 for ≥1 year.
**Seale** [76] **UK**	Compare the language of men and women with cancer in research interviews and online support groups	Internet (information and/or support)	cancer - prostate (men) and breast (women)	IV 52 men 47 women, OP 900 men, 153 women, Total 952 men, 200 women	descriptive / interpretive	interview sample details n/r; online postings sample details n/r; Ziebland 2004 (cited by authors) reports interview sample details for 49 men and 37 women as respective mean age 62 yrs (range 51-83) and 44 yrs (range 19-75)
**Seymour-Smith** [20] **UK**	How men and women negotiate their identities as members of cancer self-help groups	support group (face-to-face)	cancer - testicular (men) and breast (women)	IV 4 men 7 women	discourse analysis	men aged 26-31, women aged 33-64 yrs; all White UK; men's sample includes 1 group leader.
**Smith 2002** [77] **USA**	Views of African American men with prostate cancer who do not use the Man to Man support group	support group (face-to-face)	cancer - prostate	FG 4 men	descriptive only (supplement to quantitative study)	all African American; all members of '100 Black Men' organisation.
**Sullivan** [78] **USA**	Comparison of communication practices of men with prostate cancer and women with ovarian cancer supporting each other online	Internet (information and/or support)	cancer - prostate (men) and ovarian (women)	OP 176 men (616 postings) 134 women (1256 postings) (not extracted re: HCPs and others)	descriptive/interpretive	sample details not known (due to methods)
**Trapp** [79] **USA**	Men's preferences for cancer support groups	support group (face-to-face)	cancer - any	IV 5 men	descriptive/interpretive	various cancer types (2 melanoma, brain cancer, lymphoma, leukemia); various stages (2 metastatic, 1 stage III); 4 had previous cancer diagnosis; aged 30-69 yrs (30, 62, 62, 64, 69); all Caucasian USA; urban; majority highly educated (1 high school, 2 graduate, 2 postgraduate); all had been employed (some retired/unable to work); 2 married, 2 single, 1 widowed
**Vanable** [80] **USA**	Views of men with HIV who have sex with men about sexual risk reduction programmes, to develop a tailored intervention	various	HIV/AIDS	IV 21 men, FG 31 men, Total 52 men	descriptive/interpretive	mean 8 yrs since diagnosis; 50% reported undetectable viral load; mean age 41.4 yrs (sd 8.1, range 24-63, “mostly middle-aged”); majority Caucasian (61% Caucasian, 33% African American, 6% other); 48% employed, 48% unemployed; mean monthly income $1023 (sd 699); all men who have sex with men; 33% cohabiting, 19% relationship but living apart, 48% “did not have a primary partner”; mean 4.8 sexual partners in past year (sd 10.9).
**Wallace** [81] **USA**	Psychosocial needs of men with prostate cancer	various	cancer - prostate	FG 16 men (2 groups)	descriptive/interpretive	mean 4.3 yrs since diagnosis (range 6 months-12 yrs); mean age 66.8 yrs (range 49-81); majority Caucasian (1 African American, 1 other); range of education (8 high school, 5 college, 3 graduate school); annual income ranged $20,000-$100,000; majority married (15 married, 1 divorced).

### Key themes

Four interconnected third-order constructs associated with men’s experiences of, and perceptions towards, SMS were identified: 1) Need for purpose; 2) Trusted environments; 3) Value of peers; and 4) Becoming an expert. Our line-of-argument synthesis comprising these constructs, summarised below, provides an interpretation of the acceptability of SMS among men with LTCs, and what may act as facilitators and barriers to access of interventions and support services.

#### Need for purpose

In order to access and continue to engage with SMS, men may need to feel that a support activity has a clear purpose and addresses an unmet need. SMS that is structured, involves some element of physical activity, offers opportunities to garner new information on self-management, or that is ‘action-orientated’ , can provide a clear purpose that is appealing to men and consistent with a predilection for problem-focused coping [41, 44, 45, 47, 48, 55, 59, 63, 72, 76, 78, 82]. Study authors often contrasted this with a presumed female preference for sharing personal experiences consistent with emotion-focused coping [44, 47, 63]. Structuring meetings around talks by invited speakers, or embracing activities such as activism and lobbying, can also provide a focus for SMS activity that is valued by men [63–65, 72, 73, 75, 83].

Men may distance themselves from SMS activities that are considered ‘feminine’ [61], such as ‘touchy-feely’ discussions [82]. Being an active rather than passive participant in SMS is preferential for many [48, 52, 53, 57, 66] and can offer a way for men to regain some control and reclaim a sense of identity which has been disrupted through chronic illness [52, 57].

“…you wouldn’t keep coming in if you were going to get nothing out of it. When we were doing the exercises we thought we were getting something out of it. Just having these talks [group discussions] is not doing a lot of good. We still want a bit back” (first-order construct) [48]

“We didn’t come just to discuss things”. (first-order construct) [41]

Constructive and purposeful discussion, for example, by being problem-focused or sharing and receiving ‘factual’ information, can be more appealing to men than ‘just talking’; offering reassurance, emotional support [49, 55] and increasing feelings of control [42].

“…men emphasised the importance of getting practical results from talking therapies in their narratives, as opposed to other forms of therapy which they conceptualised as ‘just talking’”. (second-order construct) [57]

Emotional support is, however, a valued component of SMS activity, although men may feel less comfortable than women with reporting this as a motivation for using SMS due to its incongruence with ‘traditional’ (hegemonic) masculine ideals of stoicism [23, 72, 73, 82, 83] and emotional self-sufficiency [53]. Structure and/or group activities can also allow men opportunities to ‘open up’ emotionally [41, 59, 63] by approaching emotional issues or mental health “sideways on” [53]. Men can be more comfortable when emotional support arises as a ‘by-product’ of other shared activities as opposed to it being an explicit component of an SMS intervention. Emotional support that focuses on strength, perseverance, and camaraderie [62], conveyed covertly through humour [41, 48] or supportive silence [41, 42] can also be attractive to men. Aligned with this is the need to avoid overt challenges to culturally-valued masculine ideals of independence, strength and control in talking-based activities; supporting the notion that SMS can be made more acceptable to men if it “focuses less on emotional expressiveness and more on instrumental changes and control” [57] and activities are thereby reframed as a way of demonstrating these traditional masculine ideals [52].

“One of the clear barriers to accessing self-management services was the perception among the men that they consisted *solely* of support groups that involved sharing of experiences. There was little awareness of the exercise, pain management, and educational options available”. (second-order construct, emphasis added) [23]

Seeking and accepting any type of SMS can pose threats to the identity of men who align themselves with masculine ideals embodied by independence and self-sufficiency. In these cases, men may feel the need to justify or legitimise their involvement in order to preserve their identity as a man [82]. Several studies have reported the instrumental role of family or friends in prompting men’s engagement with SMS; for example, in identifying a need for support, accompanying men when attending activities, or helping to access and navigate information [43, 52, 55, 58, 60, 63, 76, 81]. Being able to ‘give back’ when engaging in SMS can be an important way for men to legitimise their involvement and lessen perceptions of their own need or vulnerability.

“Perhaps once men establish that their primary concern is to offer help to others it may became less problematic to admit to benefiting from the group themselves”. (second-order construct) [82]

‘Giving back’ via relationships with peers or through taking on leadership roles such as committee membership, can be important for male identity and self-esteem [53, 63, 82, 83]. Adopting a ‘business-like’ approach can be particularly appealing to some [63], perhaps reflecting the ways in which LTCs can challenge men’s identities as men; for example, through loss of identity through loss of work [23, 61].

“…in addition to meeting the information needs of newly diagnosed men, the group meetings needed to offer “new” information to maintain the interest of long-term members, because their commitment to the group was often premised on continuing to learn, as well as “giving back” to newly diagnosed men”. (second-order construct) [83]

#### Trusted environments

Fostering a trusted environment where men feel comfortable and able to participate in support activities is critical for accessibility and acceptability of SMS, especially where participation has the potential to make men feel vulnerable or lacking in confidence. The clearest example of this is when interventions or group-based activities involve the discussion of ‘taboo’ topics – such as mental health, sexual function, and/or emotional expression – which can challenge masculine ideals and behavioural expectations [42, 71, 72]. In face-to-face support activities, group dynamics can promote the discussion of ‘taboo topics’. In prostate cancer support groups, for example, rational and objective discussions on functionality, rather than feelings, can legitimise a supportive and collective problem-solving group dynamic that encourages men to 'open up' about potentially difficult topics, such as erectile dysfunction [72]. This way of talking can also allow men to 'open up' to different ways of thinking about activities not usually constructed as fitting with stereotypical masculine roles such as cooking or abstaining from alcohol consumption [71, 72].

“… being chauvinistic males we tend to keep it to ourselves … But when I’m amongst people like this I feel safe and confident”. (first-order construct) [48]

Group dynamics can also work to stifle emotional expression and, in some cases, a lack of emotional sharing may in fact reflect opportunities to ‘share’ are constrained by group processes rather than an unwillingness on the men's behalf [52]. Practises such as topic-turning by facilitators can serve to discourage or “squelch” emotional talk [42, 47]. Thus, although a focus on problem-solving and the practical aspects of potentially emotive topics can represent a positive way to facilitate the discussion of potentially taboo topics among men [72], it can also be a practice employed to avoid or curtail emotional talk and listening to underlying concerns and experiences [42, 71].

“Jim was visibly disturbed by the effect of the hormone on his body, but rather than address that concern, the group moved into a discussion of financial matters, an instrumental issue”. (second-order construct) [42]

Both lay and health professional facilitators of SMS are instrumental in fostering a trusted environment for men. Healthcare professionals can play a key role in either enabling or inhibiting access and this may be particularly important in mental health conditions, where establishing a one-to-one relationship with a facilitator can be crucial before men feel willing and able to attend support groups [53, 57]. In multi-component lifestyle interventions, especially those involving physical activity, supportive and positive professional facilitators have a key role in motivating men to adopt behaviour changes and supervising activities where men lack confidence [41, 43, 48, 59, 69]. Allowing men some control over their level of involvement in interventions involving both physical activity [41, 74] or discussion-based support [72, 75] can also improve acceptability. For example, a study of prostate cancer support groups noted the value men placed on being “allowed” to listen without feeling an expectation to talk.

“Men who did not want to talk could listen without worrying about being put on the spot to say something, whereas others could comfortably share questions and comments from within the group”. (second-order construct) [72]

“I finally screwed up the courage to say something … I looked around expecting people to look shocked or disapproving. … People just nodded … and reacted like it was no big deal. After that, I was able to talk more openly …” (first-order construct) [75]

Men also value having control over their level of involvement in online forums, where some may prefer to 'lurk' rather than (or prior to) posting [49, 62, 78]. ‘Lurking’ can be a necessary step for some men in the 'opening up' process; enabling those who may not feel able to ask questions to gain some benefit from the interactions of those who are more active [49]. ‘Lurking’ may also reflect men’s desire to learn the rules of talk before actively participating [62, 78] in order to become “comfortable in knowing ‘how to’ participate” [78].

#### Value of peers

Interaction with peers is widely valued by men across a range of SMS activities. Peers are generally seen as those who are “roughly in the same boat” [50]. Differences in some social characteristics (such as age, ethnicity, class/economic background) are often transcended by a shared experience of a particular health issue and by gender [41, 51]. An assumed empathy based on experiences that are sufficiently similar can allow men a 'break' from their illness (and disrupted identities) and the ability to fall back on a degree of intuition in understanding how others feel [66, 79]. This can mean that less needs to be explicitly voiced [41, 51].

“We don't need to convolute things by asking how someone feels today because we can see it … We understand just when to laugh and sometimes when we should be quiet”. (first-order construct) [41]

For many men with LTCs, the peers they encounter through SMS activities (either face-to-face or online) provide a welcome opportunity to experience a sense of belonging and normality [53, 55, 62, 72, 78]. The validation that, as men with a chronic health condition, they could regain a male 'insider' rather than 'outsider' status appears to be important across several different health conditions (e.g. cardiac conditions [43] prostate cancer [81]).

“you can't separate support from understanding. … there's nothing more supportive to me than when someone says, “Yeah, I know” or “I understand” or “it's happened to me” … that commonality” (first-order construct) [79]

“I felt part of a … team, and really wanted to be there for other people no matter what condition I was in”. (first-order construct) [50]

A strong peer-group identity can encourage health behaviour change through a sense of team spirit, camaraderie, social commitment and obligation [41, 43, 48, 59]. Peers can also offer men a “living example” [72] of hope, optimism and inspiration that can help individuals achieve a sense of perspective, and also act as a ‘credible source’ from whom they can garner information and learn about self-management [53, 59, 66, 80]. Learning from peers by sharing self-management tips and strategies [59] or reading accounts of ‘survivor stories’ [58, 70] may be more acceptable than learning from health professionals because relationships are more equal and there is less of a feeling of being “preached at” [66, 80].

In some cases, men may value attending support interventions with those who are peers across ‘several layers’. In this way, being in the ‘same boat’ requires having multiple things in common as well as shared illness experience (for example, age, gender, sexuality or culture); something particularly evident among men living with conditions perceived to be ‘stigmatised’, such as depression and HIV/AIDS [51, 53, 68, 80]. Thus, for some men, peers and trusted environments are about taking part in activities with other men in ‘male-only’ spaces, but for others it is not. For example, prostate cancer is often described as a ‘couple’s disease’ and face-to-face support groups and online discussion forums can sometimes be made more acceptable with the participation of female partners [49, 63, 78]. Regardless, support provided from peers is seen as distinct from the support received from friends and family [46, 65, 72, 75, 79]. Being away from family and friends can allow men to share experiences without fear of ramifications and the related desire to protect friends and family from the 'burden' of their own condition and associated needs [79].

“… you have also created an enormous burden on others … I belong to the support group, because … we all understand each other. There are a few people there who are very important to me. They’re not friends. … there’s that distance. We just get together to unburden …”(first-order construct) [46]

#### Becoming an expert

Many men place a high value on receiving health information and education in order to develop their capacity to manage and ‘become an expert’ in their condition [42, 44, 45, 47, 49, 55, 58, 62, 63, 70, 72, 73, 76, 78]. Developing knowledge and expertise in SMS can also provide men with opportunities to ‘give back’ to others as a lay-educator; a role that can act as a key motivator for (ongoing) use of SMS, as described above, and have associated benefits for men’s self-identity and self-esteem [47–49, 55, 66, 72, 75, 78, 79].

“People [men] are hungry for information, what is the latest in research … People are just dying to get their hands on the latest information”. (first-order construct) [65]

“Through this process of giving support to others, these men experienced an empowering sense of meaning and accomplishment” (second-order construct) [75]

Developing self-management expertise can extend to gaining skills in navigating health services, facilitating patient-health professional interactions, and attaining partnership in decision-making [55, 73]. Knowledge can allow men to gain “currency” and “power” [55], and lead to them becoming informed consumers who can ‘shop around’ for healthcare providers and treatments.

“Consumer discourses and strategies to contest power relations with health care professionals underpinned many men’s search for prostate cancer information and their commitment to assisting other men”. (second-order construct) [73]

Opportunities to build confidence and expertise in communicating with care providers can be an attractive component of SMS; for example, through face-to-face question-answer sessions or online interactions with health professionals [42, 48, 73, 78]. Such involvement with care providers can be particularly valued when men feel dissatisfied with clinical interactions; for example, due to lack of time with health professionals and lack of power and partnership [48, 52].

“[Knowledge] not only gives you the information to feel comfortable, but also gives you the information and a tool to check the physician. Not just his reputation but also the information he is giving you”. (first-order construct) [55]

Using and sharing medical terminology and technical information can be particularly attractive to men [42, 73]. That said, not all welcome the opportunity to act in the role of ‘consumer’ of health services, instead preferring to devolve decision-making to health professionals as experts who “know their stuff” [49, 58].

“Imagine being in a fast flowing river and the guy on the bank has got half a dozen different aids to help you and he's shouting to you ‘which one do you want?’. You know, I don't care which one it is as long as…you know which one to throw” (first-order construct) [58])

Overly complex or technical information can, however, also act as a barrier to learning, provoke anxiety, and overwhelm [76]. Allowing men the freedom to learn in their own way without feeling the threat of being derided for their lack of knowledge about specific health and illness issues can improve accessibility. Complex content, style, or language can reduce the accessibility of information [73] and limit patient empowerment [49]. Information that is presented in ‘everyday language’ , can be integrated with daily life, and that is tailored to demographic characteristics that men can relate to is particularly appealing for some [67, 70]. For example, strategies or usable information on “the why’s and how you do it” [70] is preferable to standardized or general health messages that can be seen as lacking “respect for the individual and his context” [56]. Significant others can also play a key role for men in obtaining information and help protect them from feeling overwhelmed by information; for example, using “lay referral networks” or “internet-savvy” friends and family, to navigate and “filter” information resources [55, 58, 76].

## Discussion

Despite growing calls for tailored and targeted health interventions to be delivered to men [32], the existing evidence-base has not yet provided a strong steer on how best to design and deliver services to address men’s distinct needs [84]. The systematic review and meta-synthesis undertaken here points toward some key considerations in relation to the content and process of SMS that may be important in helping to optimise interventions to be more accessible and acceptable to men with LTCs.

Recent evidence has shown that the accessibility and acceptability of behaviour change interventions can be improved when the context, content, and delivery style of interventions are tailored to be in alignment with valued aspects of men’s identities [31, 85–87]. A cross-cutting theme in our synthesis was the tensions that men experienced between a perceived need to fulfil roles and obligations linked to their identities as men, and acceptance of living with and needing help to manage a LTC that could potentially threaten those identities. It is clear that the physical and mental impacts of living with a LTC can pose significant challenges to men’s masculinity; a theme that has been recognised elsewhere as a “loss of self” [88] as men try to renegotiate and recapture aspects of masculine identity they feel have been lost through chronic illness [89, 90].

Our findings are in line with a recent broader review of the role and effectiveness of SMS in LTCs [91] which points toward the ‘biographical disruption’ LTCs can have on an individual’s ‘normal’ life, and the need to reconstruct one’s identity by adjusting to the physical, emotional, and societal implications of illness [92, 93]. Our synthesis places this ‘disruption’ in the context of men’s gender identity and, in doing so, indicates that SMS is most likely to be successful in engaging men when working *with*, not *against*, cultural ideals of masculinity. In other words, as Hunt and colleagues have previously stated, support interventions need to engage men without being an anathema to valued aspects of their identities [31].

Here, our findings highlight the potential importance of positioning and marketing SMS interventions in ways that pre-empt or overcome potential threats to masculine identities; whilst being mindful that men are not a homogenous group and that SMS needs and preferences will likely vary amongst men and may change with the trajectory of their illness. Strategies such as demonstrating a clear purpose to an intervention and offering opportunities to maintain control and/or ‘give back’ are likely to be beneficial, although such approaches should address changing needs and recognise different purpose in initial and ongoing use [91]. This links to the need for some men to tackle emotional issues ‘sideways on’, as a ‘by-product’ of other shared activities. Intimacy and emotional sharing may become hampered if made too explicit a (initial) goal of support activities. Crucially, our synthesis suggests that ‘trusted environments’ afforded by online communities and peer support groups can help men to overcome cultural expectations of masculinity and enable them to 'open up' emotionally.

Peer support can offer men a sense of belonging and community and was widely reported to help men adjust and come to terms with their health problems. Consistent with a recent review of weight-management programmes in men with obesity [87], being able to identify with the illness experience of others to some degree appears to be of foremost importance in determining who is a ‘peer’. However, aligned with the findings of the Football Fans in Training (FFIT) study, which attracted ‘like-minded’ and ‘like-bodied’ men who shared an interest in football and had similar physiques and levels of fitness (‘people like them’) [85], our synthesis highlighted that having multiple things in common with peers (including gender) may improve accessibility and acceptability of SMS for some men. Being around ‘people like them’ may be particularly important for men when they have a chronic problem which makes them feel that they ‘stand out from the herd’ [89]; distinct from other men and perhaps unable to ‘perform’ their masculinity in different contexts in ways which they have been accustomed to at other times in their adult life.

### Strengths and limitations of the review

The difficulty in systematically identifying qualitative studies in research databases is well recognised [36]. This is further accentuated in qualitative studies of self-management, since they are frequently not labelled as SMS, but rather are often simply referred as support groups or educational programmes. A strength of this review was the thoroughness of our search, which involved the title/abstract screening of 6330 unique records and offers a comprehensive picture of the available qualitative research. The approach adopted in the meta-ethnography did not preclude synthesis across studies of different types of intervention or support activities, but the limited amount of data and analysis reported in papers meant it was not possible to unpick the accessibility and acceptability of particular types compared with others. In addition, the synthesis is likely to have been heavily influenced by the literature on face-to-face group-based support interventions as this was the most common type of intervention/activity represented in the extant research.

Whilst the influence of culturally-dominant (hegemonic) masculine ideals was a cross-cutting theme in our synthesis, the findings need to be interpreted with caution. A body of recent work has begun to question the simplistic link between constructions of hegemonic masculinity and men’s health-care practices [94]. Evidence of the fluid and contextually-dependent nature of gender in the wider body of men’s health literature [95, 96] suggests that the studies included in our synthesis may not adequately capture the complexity of how masculinities intersect with men’s health behaviour. There is unlikely to be a ‘one-size-fits-all’ approach to gender-sensitising SMS for men. Indeed, the meta-ethnography suggested that men and women may both benefit from a particular intervention components/types (e.g. peer support, information sharing) if they have similar personal preferences and/or a shared illness experience. Although the review findings point toward some key considerations that may be important in helping to optimise interventions to be more accessible and acceptable to men, clearly, gender is not a ‘stand-alone’ variable that determines access and engagement. The factors discussed here in relation to the content and processes of designing and delivering SMS may help to improve acceptability and accessibility in certain sub-groups of men (e.g. those who adhere to hegemonic masculine ideals) but not others. Further research is required to explore the complexity of the relationships between gender and other factors known to influence access and engagement to interventions.

### Implications for future research, policy and practice

Person-centred care is at the heart of a whole system approach to LTC management [97]. In order to make SMS person-centred, findings from this review echo recommendations for interventions to be tailored to individual preferences and lifestyles [91, 98]; for men living with LTCs, this is likely to involve consideration of their identity as a man. Health professionals and service commissioners might usefully consult with male service users about how to make existing support interventions more appealing to, and congruent with, key aspects of their identities.

Gender-sensitising SMS in context (e.g. delivered in a trusted environment among peers), content (e.g. action-orientated), delivery style (e.g. a problem-solving/practical approach) and marketing (e.g. emphasis on purpose/tangible results) may yield benefits. However, health professionals need to recognise that men are not a homogenous group and that there is unlikely to be a ‘one-size-fits-all’ approach that meets the requirements of all men. Men may be willing to accept different types of interventions or activities once they feel they are in a trusted environment with peers, including ones which at the outset may have appeared to present some challenge to aspects of male identity.

Further primary research is required to examine which models of service delivery are most effective and cost-effective in providing SMS to men (and women). The complex and contextually-dependent nature of men’s engagement with self-management support interventions highlighted in this review suggests that a study drawing on realist principles [99] might be one method of analysis which might have utility. Parallel primary qualitative research is also needed to develop our understanding of what makes interventions, and their ‘active ingredients’, accessible and acceptable for men with LTCs. In particular, the self-management experiences and perceptions of men of differing age, ethnicity and socioeconomic background need to be considered. Men are a heterogeneous group, yet consideration of how these factors intersect with men’s gender identities has rarely been a focus in previous qualitative research.

## Conclusions

This qualitative systematic review and meta-synthesis aimed to determine whether SMS is accessible and acceptable to men, and explore what may act as facilitators and barriers to access of interventions and support activities. We identified four key constructs associated with men’s experiences of, and perceptions towards, SMS: 1) need for purpose; 2) trusted environments; 3) value of peers; and 4) becoming an expert. The constructs suggest that men may find SMS more accessible and acceptable when it has a clear purpose that addresses an unmet need; is delivered in an environment that offers a sense of shared understanding and ‘normality’; involves and/or is facilitated by men with a shared illness experience; and offers personally meaningful health information and practical strategies that can be integrated into daily life.

In order to overcome barriers to access and fully engage with interventions, men may need to feel that participating in SMS does not challenge valued aspects of their identities, particularly masculine ideals associated with independence, stoicism, and control. This is an important consideration for the design and delivery of future SMS interventions if they are to work to support the growing number of men living with LTCs.

## Electronic supplementary material

Additional file 1: **Second-order findings and corresponding third-order constructs of each study.** (DOCX 121 KB)

## Abbreviations

LTC: Long term condition
SMS: Self management support
PPI: Public and patient involvement.
