# Supplementary material for: The accessibility and acceptability of self-management support interventions for men with long term conditions: a systematic review and meta-synthesis of qualitative studies
Source: BMC Public Health. 2014 Nov 27;14:1230. doi: 10.1186/1471-2458-14-1230 (PMC4295235; doi:10.1186/1471-2458-14-1230)
Supplement: Supplementary file 1 — Additional file 1: Second-order findings and corresponding third-order constructs of each study. (DOCX 121 KB) [file 12889_2014_7457_MOESM1_ESM.docx]

| **Study (First Author, Year)** | **Second-order findings (main themes and ideas reported by authors)** | **Third-order constructs and overarching concepts (our interpretations)** |
| --- | --- | --- |
| Adamsen 2001[[41](#_ENREF_41)] | 1) why the men enrolled (motivation) – ‘personal conquest’, ‘victory’, physical development, dissatisfaction with body, reputation of training facility; 2) social obligation – professional-led, set meeting times, obligation towards group, comradeship via physical activity and humour and trust, understand ‘when to laugh / be quiet’, fight together against the ‘shit’; 3) well-being and bodily awareness - improved well-being, new energy, self-esteem and belief in own resources, awareness of body, different levels of ability and vary with health, lectures helpful and valued psychologist speaking *with* them not to them and use of jokes, topics (e.g. sexuality, complementary and alternative medicine) no longer taboo | *Need for purpose*  emotional vs. informational – different ways of giving emotional support ‘handle with care’;  preferences for focus and format – physical development, sense of achievement; prefer structure ‘did not meet to cry’  *Trusted environments*  ability to pitch at own level – can attend even if unable to do physical component;  group dynamics and rules of talk – ‘male-trust’ culture; embrace taboo topics via lectures; understand ‘when to laugh/be quiet’;  physical characteristics and group facilitators – professional-led, supervised, strong reputation, facilitators for facilitating talk  *Value of peers*  sense of community – identity via subculture, normalise experience, ‘in this together’, ‘break from illness’;  information, education and motivation – social obligation and commitment, and ‘fighting together’;  who is a peer – common condition transcends socioeconomic differences  *Becoming an expert*  who is expert – treated as partners by care providers |
| Arrington 2005[[64](#_ENREF_64)] | 1) Man-to-Man self-help groups are primarily used for information; 2) emotional talk is discouraged (“squelched”) by group processes: topics (avoided discussions of death, sex); topic turning (e.g. focusing on practical aspects instead of emotional), using comparisons, facilitators (including HCP). Other factors limiting emotional support/talk include: size of groups, lack of familiarity with other members, members' contact limited to meetings, possibly partner presence. | *Need for purpose*  emotional vs. informational – focus on information with emotional kept separate, although emotional may take different forms (e.g. supportive silence)  *Trusted environments*  group dynamics and rules of talk – emotional support may reflect group and rules of talk rather than individual wishes;  physical characteristics – smaller group with greater familiarity facilitate intimacy;  group facilitators – facilitators (especially HCPs) may influence views expressed;  *Value of peers*  sense of community – partner presence may limit involvement to formal contact time (also linked to older participants);  presence of women and significant others – partner presence may limit emotional support;  *Becoming an expert*  limited informed choice – group views exist concerning the condition and its management |
| Baird 2001[[66](#_ENREF_66)] | Note – analysed with respect to self-care deficit theory. 1) self-care agency – importance of health beliefs and ‘dispositions’ for adherence; 2) ‘basic conditioning factors’ influence adherence to health behaviour change, e.g. age (habits may be more entrenched); health state (wanting to avoid further illness); healthcare system (information, HCPs, other patients); family system (partners attend and reinforce messages outside contact time); pattern of living (habits inhibit change); environmental factors (heat at exercise facility may inhibit); resource availability and adequacy (financial barriers to resource access, e.g. lack of coverage by medical insurance) | *Trusted environments*  physical characteristics – financial barriers to access;  group facilitators – motivational role  *Value of peers*  information, education and motivation – motivation via camaraderie and comparison;  presence of women and significant others – partners may facilitate attendance and implementation of change  *Becoming an expert*  health literacy and desire for information – information and knowledge are important |
| Barlow 2009a[[44](#_ENREF_44)] | 1) men were more ‘critical of the course content and delivery'; 2) men and women reported similar benefits regarding self-management skills; 3) men valued informational aspects whereas women valued ‘interactive processes; 4) some men may struggle with ‘group interaction on emotive topics’ wanting factual information from ‘tutors’ rather than ‘facilitators’ of group discussion; 5) some patient valued range of conditions to offer different perspectives and reduce feelings of isolation | *Need for purpose*  emotional vs. informational – male preference for information, female preference for emotional  *Trusted environments*  group facilitators – role of facilitators in group discussion;  *Value of peers*  who is a peer – range of conditions may avoid ‘downward spiral’  *Becoming an expert*  who is expert – male preference for ‘tutors’ providing facts |
| Barlow 2009b[[45](#_ENREF_45)] | 1) men valued information exchange whereas women valued ‘emotional and social interaction | *Need for purpose*  emotional vs. informational – male preference for information, female preference for emotional; reciprocity and legitimising use – male preference for information exchange |
| Bedell 2000[[79](#_ENREF_79)] | Central theme of ‘a reasonably stable base’; section relating to support groups: 1) ‘people I feel I can lean on’ - informational support and an ‘outlet’ for emotional sharing; support group understand each other; want to protect family and friends from negative emotions | *Need for purpose*  emotional vs. informational – both informational and emotional are valued and can happen together  *Trusted environments*  group dynamics and rules of talk – being ‘allowed’ to vent  *Value of peers*  presence of women and significant others – protect family and friends and value the separation |
| Bell 2010[[46](#_ENREF_46)] | 1) content of group meetings: metastatic (women's) focuses on emotional sharing whereas colorectal (mixed sex) is emotionally ‘neutral’ and moves toward ‘safer’ topics, gender effects may be ‘flattened’ and not meet needs of men or women (some women wanted more ‘intimate atmosphere’ and ‘buddies’ outside group’; Chinese group had wide ranging topics (including practical aspects, communication issues); 2) commonalities between groups: similar perceived benefits (information, acceptance, understanding); motivations vary with treatment stage (initially more focused on information, later friendship and ‘give back’ and support others | *Need for purpose*  emotional vs. informational – male preference for information, female preference for emotional;  reciprocity and legitimising use – importance of ‘giving back’;  changing needs – needs change with stage of condition  *Trusted environments*  group dynamics and rules of talk – avoidance of emotional topics  *Value of peers*  sense of community – some women want contact outside formal group;  who is a peer – mixed sex groups may not meet needs of all members  *Becoming an expert*  who is expert – women value personal experience |
| Bourke 2012[[42](#_ENREF_42)] | 1) motivations for taking part – return to physical activity, ‘give back’ to staff and future patients; 2) supervised group design – encouraged motivation and 'male only' space away from partners; 3) social interaction - felt ‘safe and confident’ around men with ‘similar’ condition, would prefer longer duration; 4) home-based exercise – more challenging as distracted by competing priorities; 5) diet aspect - helpful and valued information but difficult to adhere to; 6) future participation – requires that intervention be viewed beneficial by self and feedback from exercise specialist, prefer group lifestyle program to peer support focused on talking; 7) exercise beyond the intervention – barriers to access including confidence and cost; 8) disease recurrence - psychological benefits (reduced anxiety and fear); 9) communication with HCPs - dissatisfaction with some; 10) benefits and drawbacks of taking part - valued goal setting, improved physical and psychological well-being but not improved urological side-effects | *Need for purpose*  emotional vs. informational – valued information around diet;  preferences for focus and format – prefer lifestyle intervention to ‘just talking’;  reciprocity and legitimising use – intervention must be viewed as beneficial to use; giving back to healthcare professionals and future patients;  changing needs – competing commitments challenge implementing behaviour change;  *Trusted environments*  physical characteristics – dedicated venue encouraged motivation; cost of gym and lack of supervision may act as barriers;  group facilitators – value exercise being supervised  *Value of peers*  sense of community – value being around similar others; interaction limited to group and would prefer longer involvement;  presence of women and significant others – prefer male-only and without partners  *Becoming an expert*  who is expert – value professional input, dissatisfied with pre-existing HCP experience; |
| Broom 2005[[63](#_ENREF_63)] | Note – describes as three themes (empowerment, control, risk) yet presents as five sections. 1) the Internet and control - information increases power and control over disease and decision-making but partly depends on HCP responses; 2) the Internet and empowerment - information allows patient to 'do something' rather than 'being told what to do’ however information may overwhelm and may help process past decisions even if ‘too late’ to influence decision; Internet can enable other roles (e.g. helping others, taking on support group leadership roles); 3) the Internet and the patient's role – empowering effect of information may be limited by HCP strategies to 'reclaim the consultation model', financial ability to choose provider, individual ability to access and comprehend information, time to make a decision; 4) trust and uncertainty - some patients are suspicious of Internet and value HCP as expert, rejecting consumerism; 5) masculinity and risk management – online setting enables some men to 'open up' and discuss sensitive topics by offering 'anonymity' and allowing different levels of involvement whereas others felt suspicious of online setting | *Need for purpose*  emotional vs. informational – information helps to regain control, tackle condition as problem to be solved  *Trusted environments*  ability to pitch at own level – opportunity to ‘lurk’ can help to ‘open up’;  physical characteristics – some men are suspicious of online setting  *Becoming an expert*  health literacy and desire for information – information may overwhelm;  who is expert – information helps to process treatment decisions and become empowered expert involve in decision-making however this is influenced by HCP responses; some men reject the consumerism role and value HCP as expert;  men may use multiple interventions in combination (e.g. information informs support groups);  limited informed choice - consumerism may be limited by financial ability to ‘shop around’ and barriers with technology / health literacy |
| Chambers 2012[[73](#_ENREF_73)] | 1) group identification - group identity based on shared experience of long-term condition (despite some variation in stage of progression), sense of ‘being there’ for others and camaraderie; 2) acceptance of diversity - differences described in positive way, enhancing group experience, all show ‘respect’ and listen to each other; 3) peer learning - learn coping strategies through sharing with others and considering different perspectives; 4) acceptance of disease progression - contact with others with more advanced disease was confronting but could offer encouragement and reassurance, ‘synergistic’ with nature of intervention (acceptance-based) | *Need for purpose*  reciprocity and legitimising use – able to ‘be there’ for others  *Trusted environments*  group dynamics and rules of talk – show core values (e.g. respect)  *Value of peers*  sense of community – sense of belonging via group;  comparison, meaning and adjustment – confrontation can be positive, and intervention promotes adjustment and meaning;  information, education and motivation – learn about coping from peers;  who is a peer – identify with others across different prognoses  *Becoming an expert*  who is expert – learning through personal experience |
| Chenard 2007[[74](#_ENREF_74)] | Central themes of 'striving for normalcy' and ‘the role of social support’; findings relating to support groups: social support is an essential part of self-care via normalising, stigma management and ‘affirming’ social networks where disclosures are not required; limiting social support to HIV/AIDS-related environments ‘assured a level of safety’ and ‘allies’ | *Trusted environments*  group dynamics and rules of talk - ‘assured a level of safety’ via being with peers;  *Value of peers*  sense of community – importance of normality, reduced stigma and ‘affirming’ social interactions, also intuition and shared understanding;  who is a peer – peers through shared condition and sexuality |
| Corboy 2011[[59](#_ENREF_59)] | Note – analysed with respect to behavioural model of health service use. 1) predisposing characteristics – age (older men perceive symptoms as part of ageing therefore less in need of support), social structure (varied awareness of services, some disappointment with HCPs, helps to know someone ‘in the [healthcare] system’), health beliefs (some issues of stigma and embarrassment, reservations about effectiveness of support groups, including reliability of information, but male reluctance to discuss health is a myth); 2) enabling resources – personal/family (can enable or be a barrier), community (rural can influence travel and delays in receiving appointments but greater barriers may be beliefs and fears around privacy); 3) need for care – perceived (independence and stoicism as barrier, minimise and downplay problems), evaluated (HCPs can find it hard to judge support needs and needs can change with time) | *Need for purpose*  emotional vs. informational – some reservations about emotional sharing and that talking may be unproductive, but willing to discuss health; male reluctance to access emotional support due to stoicism;  reciprocity and legitimising use – family/friends may influence access;  changing needs – existing support may influence access; older men perceive symptoms as part of identity therefore less need to address; rural life may influence access via logistical barriers or influence on beliefs about services and concerns around privacy; need may vary back and forth rather than linear  *Trusted environments*  group facilitators – HCPs may act as barrier to service use  *Becoming an expert*  health literacy and desire for information – knowing ‘insiders’ may help to navigate services;  who is expert – information should come from ‘qualified’ person rather than patients;  limited informed choice – lack of awareness of services as barrier |
| Cramer 2013[[60](#_ENREF_60)] | 1) isolation and social benefits - men often isolated, valued support outside family or friends, valued ongoing support, may need one-to-one instead of group in periods of ‘crisis’; 2) value of groups and strategies for attracting men - establishing trusting relationships through one-to-one work with facilitator before joining group, providing activities (e.g. food) that allow mental health to be addressed ‘sideways on’, opportunity for leadership (via roles in the group), type of facilitators (preferences may link to socioeconomic background), importance of peers (e.g. reducing stigma, learning from each other), men-only may or may not inhibit sharing and talking openly about feelings; 3) accessing support and the role of health professionals - GPs can act as enabler or facilitator for accessing support, counselling sometimes seen as unproductive | *Need for purpose*  emotional vs. informational – some men value emotional support highly; some men do not value activities viewed as ‘just talking’ without problem-solving focus;  preferences for focus and format – activities enable tackling emotional issues ‘sideways on’; reciprocity and legitimising use – may adopt leadership roles;  changing needs – emotional support valued where lack of existing informal support and may vary with stage of condition (e.g. ‘crisis’)  *Trusted environments*  Group facilitators - value of one-to-one support before joining group and varied preferences for facilitator characteristics; HCPs may act as barrier or enabler to service use  *Value of peers*  sense of community – reduce stigma; value ongoing support rather than time-limited;  information, education and motivation – peers offer accessible learning;  presence of women and significant others – male-only environments may or may not inhibit emotional sharing; value support outside friends and family |
| Dickerson 2006[[98](#_ENREF_98)] (linked women’s study); Dickerson 2011[[47](#_ENREF_47)] | Sex comparisons (Dickerson 2011): men focus on problem solving and women focus on ways of living with condition; women use family/friends more for accessing information; men want power in HCP interactions whereas women want to verify decisions; both value patient stories for symptom management and expectations but men primarily use for functional aspects (symptom management, adverse effects, treatment) whereas women use primarily for support, advice, encouragement.  Men’s data set (Dickerson 2011): overall pattern - ‘cancer diagnosis as a problem to be solved’; five themes: 1) seeking information for decision making and treatment plan; 2) retrieving information to facilitate HCP interactions and monitor for reoccurrence; 3) evaluating information, including using views of HCPs; 4) patients’ (online) stories informing possible symptom management; 5) navigating the ‘healthcare system politics and power’.  Women’s data set (Dickerson 2006): overall pattern - ‘Internet use as assisting in discovering ways to live with cancer as a chronic illness instead of a death sentence’; five themes: 1) retrieving and filtering contextualised information using ‘Internet-savvy’ friends or family; 2) seeking hope while avoiding fear, using ‘manageable “bytes”’; 3) self-care regarding specific symptom management; 4) empowerment through providing ‘second opinion in decision making and validating treatment decisions’; 5) providing peer support. | *Need for purpose*  emotional vs. informational – male preference for information as a way to tackle problems and cope;  *Value of peers*  sense of community – ‘validate’ experience and ‘not alone’;  comparison, meaning and adjustment – can offer optimism or ‘downward spiral’; acceptance and adjustment usually via social comparison although also via information processing; hope and optimism may be important for women  *Becoming an expert*  health literacy and desire for information – ‘Internet-savvy’ friends/family may help to access and filter information; use of technical information;  who is expert – peers considered experts (although men favour for symptom management and expectations whereas women favour for support, advice, encouragement); become own experts and seek empowerment (through navigating health services, partnership in decision-making, acceptance of past decisions) but also influenced by HCP responses; evaluate credibility of information, ‘selective’ with discussion boards and care providers, use of multiple sources, use of multiple interventions (e.g. support groups as ‘catalyst’ for consumerism) |
| Eldh 2006[[81](#_ENREF_81)] | 1) ‘Participation’ viewed by men as: being an equal partner (shared decisions), accepting responsibility, being responsible, being seen in one’s context (treated as an individual with contextualised information); 2) ‘Non-participation’ viewed by men: being controlled (commands and instructions), lacking respect (not listened to or treated as individual), lacking information; 3) Men and HCPs had ‘conflicting values’ – respective emphasis on responsibility and information, and HCPs perceived themselves as more involving and individualising than perceived by patients and participant observation. | *D)evolving consumer*  health literacy and desire for information – need for individualised information that considers context, to promote understanding and translation into knowledge that can be integrated in daily life;  who is expert – some men view that HCPs may without information due to having views on certain choices being ‘correct’; patients perceive lack of power and want to be equal partners rather than controlled and not respected by HCPs;  limited informed choice – patients are expected to accept responsibility (which they may not resist) |
| Emslie 2007[[61](#_ENREF_61)] | 1) men and women may struggle to recognise and articulate mental health problems; 2) men and women value certain aspects of HCP relationships (being listened to, taken seriously, not rushed, caring, trust, rapport) but diversity within groups whether prefer ‘talking to a stranger’; 3) ‘different emphasis in communication’ with gender - men value HCP skills that help them talk whereas women value listening skills; 4) ‘male emphasis on getting practical results from talking therapies’ using problem-solving rather than ‘just talking’ | *Need for purpose*  emotional vs. informational – male preference for problem-solving and productive talk (rather than ‘just talking’);  preferences for focus and format – male preference for focus on instrumental changes rather than emotional sharing  *Trusted environments*  physical characteristics – some men and women prefer to confide in HCPs who are well known to them whereas others prefer greater anonymity;  group facilitators – HCP can be enabler or barrier to accessing intervention; men and women value key attributes in HCPs (e.g. being listened to) although men may value skills that help them ‘open up’ whereas women may have greater concerns about listener responses |
| Evans 2007[[67](#_ENREF_67)] | 1) men may be ‘proactive seekers’ or ‘passive recipients’ of CAM information – main information source is ‘lay referral’ network (family/friends, especially women, sometimes linked to proficiency with Internet especially if older); approach varies with characteristics (e.g. use prior to cancer, type and stage of cancer); some did not want to seek CAM information (feeling that NHS should signpost, lacking confidence, wary, wanting NHS ‘stamp of approval’); proactive usually view as empowering (gain control, hope) whereas passive usually view as anxiety provoking or overwhelming; some individuals change from passive to proactive; 2) patients use multiple types of evidence and from various sources (Internet and ‘traditional’) and use various criteria for evaluating CAM information and therapies – often ‘discerning’ rather than ‘wholesale acceptance’; want belief and trust in therapies and providers (e.g. NHS ‘stamp of approval’). | *Need for purpose*  changing needs – approach to seeking CAM information may vary with type of LTC (e.g. rarer), stage of illness (e.g. unresponsive to 'conventional' medicine), time (moving from passive to active)  *Becoming an expert*  health literacy and desire for information – ‘Internet-savvy’ friends/family may help to access information (and this may have intersection with age, although not necessarily gender), therefore some prefer ‘traditional’ sources; information may ‘overwhelm’;  who is expert – men value both peers and HCPs as experts and may want HCP ‘stamp of approval’ despite dissatisfaction with HCPs; some men evaluate credibility of information by consulting multiple sources; not all men wish to become active consumers;  limited informed choice - efforts to become empowered may be limited by HCP response |
| Ferrand 2008[[43](#_ENREF_43)] | 1) social motive – ‘convivial team atmosphere’ (men and women value friendship, talk and exchange with others, being respected and valued); 2) social motive – feeling understood and well-supported (medico-sporting educators offer information exchange; women value emotional support, friendship, encouragement and group activity from peers whereas men value information, skills, strategies; few men or women mentioned family support); 3) social motive – ‘pleasure in a group’ (women value group and doing something for self rather than family); 4) psychological motive – well-being (valued by both but women also about bodily awareness); 5) psychological motive – health (valued by both but women focus on flexibility, mobility, pain and men focus on stopping weight gain, increased fitness, energy, delays ageing); 6) psychological motive – body image (found in women only, linked to confidence) | *Need for purpose*  emotional vs. informational – male preference for information, skills, strategies; emotional support takes different forms (nurturing and warmth in women vs. encouragement in men);  preferences for focus and format - group activity offers opportunity for socialising and emotional sharing; men and women reported improvements in physical health and wellbeing (women reported flexibility, mobility, pain whereas men reported fitness, energy, weight loss, delayed ageing)  *Trusted environments*  group facilitators – provide support for physical activity; value information exchange rather than power imbalance  *Value of peers*  sense of community – think as ‘we’ and can share experiences; view as friends;  information, education and motivation - men value tips from peers; supportive and sense of ‘team’ |
| Galdas 2012[[68](#_ENREF_68)] | 1) food – diet is strongly linked to family practices and gendered division of domestic labour and a social activity at the Gurdwara (Sikh temple) therefore changes hard to maintain without family/friend support; 2) exercise – some prefer walking and socialising with others to formal exercise regimen with ‘strangers’, education and monitoring in programme can provide self-efficacy for self-led but some still lack confidence without HCP guidance; some men who did not access intervention are already independent; 3) faith and religion – predetermination and external locus of control shape health beliefs that could act as barrier to access, however some diversity of views | *Trusted environments*  group dynamics and rules of talk – involvement of family and friends may help to implement behaviour change, especially in collectivist cultures;  physical characteristics – some prefer formal supervised setting to provide confidence for physical activity;  group facilitators - some value supervised setting to provide confidence for physical activity  *Value of peers*  information, education and motivation – some prefer solitary walking or walking with friends to exercising with ‘strangers’;  who is a peer – some view other patients as ‘strangers’ |
| Gibbs 2005[[58](#_ENREF_58)] | 1) hegemonic masculinity influences men’s perceptions of services: perceive services as solely about support groups and emotional sharing (unaware of exercise, education, pain management); emotional sharing viewed as feminine and sign of weakness with homosexual connotations (use of sexist and sexual references in humour); however severity of condition and its impact (including ‘emotional needs’) may ‘override hegemonic masculinity’; 2) hegemonic masculinity influences men’s perceptions of self: condition compromises hegemonic masculinity; men may have multiple conditions each requiring identity adjustment; treating condition as a technical problem to be solved helps regain control, fitting with hegemonic masculinity | *Need for purpose*  emotional vs. informational - male preference for problem-solving as offering control; group membership (both condition and access of services) carries implications about identity with emotional sharing viewed as feminine/gay activity and a sign of weakness;  changing needs – men may have multiple conditions, each with individual needs and implications for identity; changing severity of condition may override competing views that act as barriers  *Becoming an expert*  limited informed choice - lack of knowledge and misconceptions about types of services available may prevent access |
| Gibbs 2007[[23](#_ENREF_23)] | 1) work as a conceptual barrier – work is an indicator of health and ability to fulfil roles therefore men do not identify as needing services whilst still able to work; 2) work as a structural barrier – some men lack time and energy to access services due to work commitment however the greater barrier is ‘placing work as a priority’ rather than employment type (e.g. rural, self-employed); 3) work as a socio-cultural barrier – men prioritise work over health rather than assigning value to health/services however this varies with severity of condition; priorities are similar across culture and employment types but less work priority for younger men (work is temporarily disrupted) and older men (view as part of ageing) whereas middle years face ‘greatest obligation’ (different hegemonic masculinities at different stages in life course) | *Need for purpose*  changing needs – perception of ‘needing’ services varies with severity of condition (perceive as unrequired when able to fulfil roles); work is a barrier to access for some men but this may reflect sociocultural factors rather than purely ‘structural’ barriers (time, energy); work as a barrier varies more with age than culture or employment type; greater accessibility and acceptability when younger or older and especially once retired whereas middle years carry ‘greatest obligations’ |
| Gooden 2007[[65](#_ENREF_65)] | 1) information support 'facts about the disease' - personal experience was exchanged but  professional opinion 'took precedence'; literature was cited by both sexes but women gave short references and men provided detailed technical summaries; evidence-based practice and scientific rigour was promoted; alternative practices were considered in women's but not men's; informed choice and consumer perspective promoted, especially in men's; 2) information support 'dealing with effects of the disease' - patients relayed own experiences but women summarised facts whereas men provided detailed medical information (including impact on bodily functions and sexual impacts); both discussed disease site and shared strategies but men focused on function and women on appearance; 3) emotional support 'coping philosophy' – both sexes offered 'wisdoms'; both used humour but men's use was lengthy and way to address emotions whereas women's was more incidental; 4) emotional support 'nurturing and expressing' - women used overt emotional expression whereas men's often implied or 'intellectualized'; women encouraged through affection and nurturing whereas men promoted strength, perseverance and camaraderie; both challenged other members and set boundaries for communication; both expressed anger and dissatisfaction with HCPs and services; both had sense of connection and 'the group'; both showed some telephone contact outside online group | *Need for purpose*  emotional vs. informational – both sexes use similar proportion of emotional and informational support but communicated in different ways (e.g. use of technical approach and humour in men) and with different types of encouragement (strength, perseverance, camaraderie in men vs. nurturing and affection in women); emotional and information may be separable;  reciprocity and legitimising use - exchange on information and strategies found with both sexes  *Trusted environments*  group dynamics and rules of talk - both sexes can challenge other members and set ‘rules of talk’; both discussed taboo topics but men had more emphasis on function than appearance and physical/practical than emotional; men used humour at length, as a form of coping and way to address emotions whereas women’s use was more 'incidental';  group facilitators - dissatisfaction with HCPs and services found in both sexes  *Value of peers*  sense of community – both sexes gain sense of community; both showed evidence of telephone contact outside online discussions;  presence of women and significant others – support from patients is different to family/friends;  *Becoming an expert*  health literacy and desire for information - consumerism promoted in both although male emphasis on medical technical jargon and female emphasis on strategies for interactions with HCPs;  who is expert - both sexes view HCP as superior to lay when concerning knowledge of disease;  limited informed choice - informed choice and consumerism promoted but within conventional treatments, although women were more 'open-minded' to alternatives (possibly reflecting absence of HCPs in women’s forum) |
| Gray 1996[[48](#_ENREF_48)]; Gray 1997a[[54](#_ENREF_54)] (linked men’s study); Gray 1997b[[53](#_ENREF_53)] (linked women’s study) | 1) information - highly valued by both but primary for men and for women it overlaps with emotional; men value from HCPs, women value from peers; 2) emotional support – intimacy is ‘cornerstone’ for women vs. secondary for men, developing over time; 3) group organisation and structure – men have business-like task-centred approach whereas women's focus is mutual support; 4) advocacy and lobbying - men are more keen for advocacy; 5) family participation - men are more keen for others to be involved (e.g. HCPs) whereas women want to retain intimacy with other women; 6) community - men want to involve others whereas women want their own space; 7) valuing laughter - women valued laughter whereas not mentioned by men; 8) encountering death - women considered how to handle death of members whereas not mentioned by men; 9) sexuality - men reported more sexual concerns but this may reflect conditions or emphasis on and acceptability of discussing sexuality | *Need for purpose*  emotional vs. informational – both sexes value information but for women emotional is the ‘cornerstone’ and there is greater overlap between the two whereas for men emotional develops over time  preferences for focus and format – men value activities and tasks (e.g. presentations, focus on business and advocacy) and these can offer opportunity for emotional sharing  *Trusted environments*  group dynamics and rules of talk – men discussed sexual aspects, women discussed death and valued laughter;  physical characteristics – women’s valuing of intimacy is better facilitated by smaller groups and men’s preference for information better suited to larger groups  *Value of peers*  presence of women and significant others – women wanted to retain their own space whereas men welcomed family and HCPs  *Becoming an expert*  who is expert – men valued information from HCPs whereas women valued from peers;  limited informed choice – men may favour having a group agenda targeting for example advocacy and lobbying |
| Harris 2007[[62](#_ENREF_62)] | Individual counselling: 1) benefits - reduced isolation, especially important at key times; accept responsibility; explore and vent emotions; signpost to other support; problem-solving; information including referrals and health strategies; safe and respectful environment; discuss sensitive and private issues not appropriate for group setting; 2) counsellor qualities – various including non-judgmental, empathetic, knowledgeable conveying expertise (especially in times of crisis) whilst having ‘egalitarian relationship’; 3) therapeutic alliance - various including egalitarian, client involvement, accomplish goals, feel comfortable with counsellor; 4) issues addressed - several including practical, health behaviours, emotional, existential, identity.  Peer support: 1) benefits - reduced isolation, camaraderie and friendship, ‘not alone’, physical resources (e.g food), skills (e.g. social skills), vent emotions, distraction, information; 2) relationships - equal power, increased openness, ‘same boat’, ‘don’t judge’, clear communication through shared experience; 3) role models - peer support workers who ‘truly understand’ and can signpost to other resources | *Need for purpose*  emotional vs. informational – some men need an emotional outlet but some place greater importance on practical and problem-solving rather than unproductive talk;  changing needs - greater need for support early after diagnosis, in times of crisis and due to social isolation  *Trusted environments*  group dynamics and rules of talk – peers do not ‘judge’;  group facilitators – men value characteristics such as non-judgmental, empathetic, knowledgeable whilst egalitarian  *Value of peers*  sense of community – peers offer feeling that ‘not alone’ and clear communication via shared experiences and ‘truly understanding’ due to being in the ‘same boat’;  information, education and motivation – men value information exchange with peers and fostering development of social skills  *Becoming an expert*  who is expert – men value equal power amongst peers, may need ‘expert’ counsellor in times of crisis but still egalitarian; different support activities can be complimentary and meet different needs and may involve signposting between activities |
| Iredale 2007[[80](#_ENREF_80)] | 1) information received - some dissatisfaction with amount of information provided; some reported helpful HCPs who provided information and support; 2) gender-specific information – many wanted, especially younger men (although the age observation came from quantitative data) | *Becoming an expert*  health literacy and desire for information – need to tailor information to gender and age;  who is expert – some HCPs offered useful information |
| Kendall 1992[[78](#_ENREF_78)] | 1) intimacy - need for connection, ‘closeness’ and ‘unity’ is primary reason for access, develops via activities (e.g. ‘sharing’); 2) group process – ‘gay bonding’ (importance of being around other gay men, talking about gay issues, identity), ‘being realistic’ (rather than in denial), ‘confronting group members in supportive ways’ (building honesty and intimacy), ‘promoting a wellness orientation’ (intimacy as a source of wellness); 3) group structure - closed groups are the most intimate (build trust through consistency of members), open groups can be ‘bonded’ provided enough structure and consistency, smaller groups promote wellbeing and time to deal with everyone’s issues, different levels of groups are needed to meet different needs (e.g. newcomers’ crisis group, intermediate group, advanced level group dealing with ‘hard core’ issues including existential), there are valued leadership qualities (e.g. active, facilitate); 4). meaning – gained through ‘reassessing life priorities’ (meaning of HIV in their lives) and building a caring community (spiritually bound community as ultimate goal of group) | *Need for purpose*  emotional vs. informational – preference for ‘connection’ over information;  preferences for focus and format – activities not necessary for all men to ‘open up’ although may take time for deeper feelings to be voiced;  changing needs- different focus of groups may be needed at different stages of condition (e.g. crisis); intimacy particularly important when lacking in own network  *Trusted environments*  group dynamics and rules of talk – important to ensure time for everyone issues to be addressed;  physical characteristics - small closed groups facilitate trust, sharing and intimacy;  group facilitators – leadership qualities (e.g. active, facilitative) are valued  *Value of peers*  sense of community – importance of being around others and feeling ‘part of life’; ‘spiritually bound community’ as ultimate goal;  comparison, meaning and adjustment – groups help to reassess ‘life priorities;’  who is a peer – value 'double' peers (HIV/AIDS and gay) |
| Kronenwetter 2005[[71](#_ENREF_71)] | 1) peer community (which included involvement of family) was the most highly valued component providing shared activities, support, socialising, ‘connection, and ‘belonging’; 2) ‘spirituality’ linked to the intervention was valued by some men; 3) ‘value’ included ‘emotional reactions’ to the intervention (e.g. optimism, hope, ‘fighting spirit’, reduced anxiety, and some ‘negative’ reactions), feeling ‘healthier’ and energised, becoming more emotionally available | *Need for purpose*  emotional vs. informational - men value ‘doing’ although talking can be included in this;  preferences for focus and format – yoga and eating together valued as part of community activities;  changing needs – intervention was long-lasting (continuing for several years)  *Trusted environments*  physical characteristics – valued community setting;  group facilitators – supportive for undertaking behaviour change  *Value of peers*  sense of community – sense of belonging as part of community; peer ‘community’ highly valued, including community activities (e.g. yoga, eating, clearing up shared space);  comparison, meaning and adjustment - gain hope, optimism, fighting spirit, reduced anxiety, and sense of ‘something bigger than ourselves’ through peer community;  presence of women and significant others - significant others involved as part of wider group |
| Martin 2013[[77](#_ENREF_77)] | 1) goal setting – some value, some struggle (e.g. if not done in work environment, if younger, if depressed, if ‘lack’ goals) and need examples; 2) information – some want it not to be too technical, welcome ‘how to’ strategies, want tailored and ‘contextualising’; 3) survivor stories – want examples they can relate to (e.g. not ‘too American’, not all professionals, some younger); 4) psychological health – some expressed few emotional impacts, some valued addressing fear of recurrence, importance of survivor peers, listening and sharing, role of facilitators valued; 5) timing – mainly wanted intervention sooner, earlier in treatment | *Need for purpose*  emotional vs. informational - some found psychological benefits through addressing fear of recurrence via information;  preferences for focus and format – many men favour structure and activity, including goal setting, but may struggle if younger, low mood, or lack goals therefore need examples and skills training;  changing needs – may benefit from earlier intervention;  *Trusted environments*  ability to pitch at own level – some welcome opportunity to share whereas others do not feel comfortable asking questions;  group dynamics and rules of talk – relaxed, sociable environment facilitated sharing, contrary to the ‘masculine expectation’ that ‘men do not openly discuss’;  group facilitators - important for men ‘opening up’  *Value of peers*  sense of community – provides sense of ‘universality’ that ‘one is not alone’;  comparison, meaning and adjustment - comparisons with roles models, survivors and those in harder positions offers helpful perspective;  information, education and motivation - learning could occur through survivor stories;  who is a peer – some wanted ‘contextualised’ information that they could relate to by having survivor stories that shared characteristics such as country, culture, socioeconomic background and age  *Becoming an expert*  health literacy and desire for information – men welcomed ‘how to’ strategies (including practical information on diet and exercise), some wanted information to not be too technical, some wanted contextualised information |
| Mfecane 2011[[77](#_ENREF_77)] | 1) 'constructions of masculinity in bushbuckridge' - men have 'economic and emotional independence', are 'self-sufficient', 'stoical rather than expressive' and the 'dominant partners' therefore not content with mixed sex groups or sharing with women; consumption of alcohol and traditional medicines demonstrates masculinity; 2) 'support groups' – view facilitators as educators (experts) and patients as students; some expert patients are viewed as role models and part of healthcare team; 'top-down approach' to effect 'responsible behaviour'; traditional health beliefs condemned, 'convert[ing]' patients may involving being 'chastised' by staff and patients; prescribe solutions rather than listen to concerns; 3) 'therapeutic citizenship and masculinity' - praise support group and benefits (information, knowledge, acceptance of diagnosis, resist stigmatisation, combat hopelessness, connect with peers) but feel obliged to abandon previous identity, therefore 'ambivalence' (enabling for coping with condition but disabling to abandon masculine lifestyles and adopt non-masculine ones) | *Need for purpose*  preferences for focus and format – programme structured around information and education as men did not feel comfortable with emotional expression;  reciprocity and legitimising use - motivation for attending was entitlement to healthcare  *Trusted environments*  group dynamics and rules of talk - emotional topics were considered taboo, group focus on prescribing solutions rather than listening to concerns;  group facilitators – some men were not receptive to advice from female facilitators  *Value of peers*  sense of community – men were able to ‘connect’ with others, feeling reduced stigma;  comparison, meaning and adjustment – group facilitated acceptance of diagnosis, and psychological benefits of combatting hopelessness and greater optimism;  information, education and motivation – expert patients viewed as knowledgeable, unlike other group members; expert patients were critical for modelling health behaviours because seen as threats to previous masculine identity;  presence of women and significant others – men found mixed sex groups difficult  *Becoming an expert*  who is expert – men viewed facilitators as educators (experts) and patients as students although some expert patients were viewed as role models and part of healthcare team;  limited informed choice – group attendance was a condition of receiving antiretroviral therapy, and had clear agenda including ‘conversion’ to a ‘responsible’ lifestyle and public disclosure of HIV status, therefore prescribed lifestyles and treatments rather than informed choice approach |
| Oliffe 2008[[56](#_ENREF_56)] | 1) ‘micro level influences: leadership as the lynchpin to meeting diverse individual needs' - leaders/committee organise meetings and speakers; committee preferable to avoid burden; realistic workload and leadership succession needed to ensure sustainability; leaders engage new members and establish rapport and camaraderie; leaders offer ‘new’ information to maintain interest of long-standing members who seek continued learning as well as ‘giving back’ to new members; 2) ‘meso level influences: emancipation or affiliation' – tensions exist about whether groups should build individual identity or collective power and retain autonomy or adopt standardised format; 3) ‘macro level influences: insufficient capacity for activism' - sustainability threatened if limited resources are redeployed from local to global activities | *Need for purpose*  reciprocity and legitimising use – ‘giving back’ motivates continued involvement; leadership roles are often but not always positive due to challenges such as burnout and burden (especially with worsening health);  changing needs – new members have different information needs  *Trusted environments*  group facilitators – men prefer peer-led but with various qualities (e.g. ability to engage and new members and provide older members with ‘new’ information); survival of group can hinge on leadership qualities  *Value of peers*  sense of community – vary as to whether view as wider (prostate cancer) community or local group, and whether affiliate with professional organisations  *Becoming an expert* (information preferences and health literacy)  limited informed choice – views vary on extent to which groups should be concerned with wider political activities and lobbying |
| Oliffe 2010[[52](#_ENREF_52)] | 1) ‘living examples of healthy men’ - importance of shared diagnosis for connection and normalising; some men value information exchange over camaraderie and friendship; men often value needs-driven activity-based meetings; men value living examples who offer comparison, reassurance, self-reflection, hope, optimism; roles change with time whereby men later ‘give back’ to other group members; 2) ‘mixing health and illness messages’ – discussion move ‘seamlessly’ between health and illness information; presentations and peers encourage adoption of healthy lifestyle; involvement of partners can encourage discussion of emotions; 3) ‘tailoring trajectory and problem-specific information’ – main goal of newcomers is empowerment for informed treatment choice; important to have ‘expert’ men and opportunity for discussion of specific treatment options in small groups; value sharing strategies and joint problem-solving; have permission to discuss taboo topics because discussed by other men and focus on problem-solving rather than emotional experience | *Need for purpose*  emotional vs. informational – emphasis on problem-solving and practical strategies rather than emotional-sharing; intimacy and friendship valued by some;  preferences for focus and format - preference for 'needs-driven, activity-based' meetings consistent with 'masculine ideals';  reciprocity and legitimising use – men value ‘giving back’ to newer members;  changing needs – information needs and role in group varies with stage of condition  *Trusted environments*  group dynamics and rules of talk – focus on problem-solving enables discussion of taboo topics; involvement of partners encourages discussion of emotions  *Value of peers*  sense of community - shared diagnosis and having ‘gone through the same’ offers belonging and the feeling that 'not alone';  comparison, meaning and adjustment - psychological holistic benefits include hope, optimism, feeling less scared and reconsidering priorities (e.g. living, rather than sexual aspects)  information, education and motivation – role models offer 'living examples' that enable comparison and encourage adoption of healthy behaviour;  presence of women and significant others – vary as to whether want to involve family and friends; some value that peers are different and want to protect significant others  *Becoming an expert*  who is expert – peers are viewed as experts |
| Oliffe 2011[[55](#_ENREF_55)] | 1) ‘numbers and measures as the foundation of prostate cancer literacy’ – across illness trajectory men are able to exert control and partnership in decision-making through information; information focused on treatment options and side effects, conveyed using ‘group dialect’ linking biomarkers and probabilities; 2) ‘group information processing’ - information stimulated group discussion and could provide hope; access to expert speakers gave current information and opportunities to gain confidence interacting with HCPs; men had varied involvement (some listened rather than talked); 3) ‘shopping around’ – knowledge given to navigate health systems, exercise consumer rights to choose doctor and treatment, including CAM; specific strategies given for conveying ‘consumer identity’ in HCP consultations; information presented as agenda-free (although certain active options may be favoured) and not rushed, unlike clinical appointments | *Need for purpose*  emotional vs. informational - focus on information and problem-solving rather than emotional aspects; exert control via knowledge and practical strategies  *Trusted environments*  ability to pitch at own level – men had varied involvement (some listened rather than talked)  *Value of peers*  comparison, meaning and adjustment –comparisons offered hope and optimism  *Becoming an expert*  health literacy and desire for information - information conveyed using numbers and ‘group;  who is expert - peers valued as expert and trustworthy ; HCPs valued as experts and groups offered opportunity to ask HCPs questions and gain confidence with interactions;  limited informed choice – groups promote consumerism, entitlements, informed choice and empowerment; information presented as agenda-free (unlike clinical appointments) although more active options may be favoured above, for example, watchful waiting or active surveillance |
| Ramachandra 2009[[72](#_ENREF_72)] | 1) men were less interested in ‘psychological aspects’; 2) both sexes gave altruism and gratitude, not personal benefit, as reasons for taking part | *Need for purpose*  emotional vs. informational - men less interested than women in ‘psychological’ aspects;  reciprocity and legitimising use – both sexes may have different motivations for taking part in research (e.g. ‘gratitude’ to care providers) that do not transfer to real-world implementation |
| Sandstrom 1996[[57](#_ENREF_57)] | 1) 'becoming involved in a support group' - men became involved for different reasons (information and advice, empathy and emotional relief, camaraderie and friendship); 2) different types of support group participation exist (long-term, brief, non-participation) - long-term usually diagnosed before HIV/AIDS awareness existed, faced high stigma and lacked access to other support, therefore valued emotional sharing, information exchange, and helping others but experienced some disbenefits (grief, 'dying out' of group, unwanted roles); brief participants usually diagnosed since increased awareness and had greater access to more support, were more interested in instrumental support (e.g. receiving and exchanging information and coping strategies) and stopped due to 'discomfort with emotional climate' (dealing with emotional instability of those experiencing different stages of adjustment and feeling that facilitators should have managed these dynamics), wanting more useful information and coping strategies (e.g. constructive talk about controllable aspects), experiencing a ‘lack of exemplars of productive coping' (not finding 'role models'); non-participants reported sufficient support from existing networks, not wanting to be confronted by their 'future' (by seeing those with worse health), being in relatively good health and that they may become involved when faced with deteriorating health (both for extra support and to 'lessen the burden' on others) | *Need for purpose*  emotional vs. informational – men wanted information and emotional relief; some distinguished the need for purposive talk;  reciprocity and legitimising use – men placed importance on receiving and exchanging information; leadership roles were not always wanted;  changing needs – emotional needs varied with informal support (less need if strong informal support) and stage of disease (greater need with crisis period and worsening health); information needs may vary with condition (for example where little information is widely available)  *Value of peers*  sense of community – some men sought friendship and wanted contact beyond formal group meetings;  comparison, meaning and adjustment – some men resisted being confronted with their future by seeing others in worse health; being around others who had not adjusted to their condition could lead to downward spiral; men could face grief and loss through groups ‘dying out’;  information, education and motivation – some wanted to exchange helpful information but found a lack of role models;  who is a peer - possible need for 'matching' groups by stage of adjustment to illness; presence of women and significant others - peers offer ability to protect and unburden family/friends  *Becoming an expert*  who is expert – peers viewed as experts;  limited informed choice - some do not want to get involved with lobbying or political activity as view that this is outside their control |
| Seale 2006[[49](#_ENREF_49)] | 1) men mainly use Internet for information whereas women mainly use for social and emotional aspects, although may use for both; 2) men more concerned with specific body areas whereas women more holistic; 3) men more concerned with treatment information and HCPs whereas women more concerned with impact on significant others; 4) women more concerned that information is untrustworthy and has potential to overwhelm or cause anxiety; 5) women show greater emotional expressivity whereas men more inhibited and use ‘concerned’ ‘embarrassed’ to convey emotion; 6) both sexes view web forums as relatively private so can discuss bodily function (and for women, privacy facilitates interactions characteristic of women’s friendship groups); 7) family and friends are sometimes responsible for information gathering from Internet (particularly in men, although this was quantitatively informed) | *Need for purpose*  emotional vs. informational - men mainly use Internet for info whereas women mainly use for emotional and social; emotional sharing may take different forms in men and women (e.g. male use of ‘concern’, ‘embarrassed’)    *Trusted environments*  group dynamics and rules of talk - problem-solving is used to avoid discussion of emotion; men’s discussion are more localised and concerned with physical aspects whereas women’s are more holistic and include distress;  physical characteristics - both sexes view web forums as relatively private; women more concerned that online information may be untrustworthy  *Becoming an expert*  health literacy and desire for information – men have more interest in medical information; information has potential to overwhelm and cause anxiety;  who is expert – both sexes needed to sift information and some used family and friends to gather and filter information |
| Seymour-Smith 2008[[50](#_ENREF_50)] | 1) 'signalling trouble' - women ‘un-problematically’ accepted researcher's positioning of them as members of self-help groups whereas men resisted this identity; 2) ‘stereotypical constructions' - men talked about stereotyped versions of self-help groups (e.g. 'touchy feely') whereas women discussed advice and support activities in non-problematic; 3) 'four functions: a matter of identity' - men foreground gender and distance themselves from stereotypical self-help groups and how they are perceived; 4) ‘“legitimate” involvement' – men ‘deny agency’ in seeking support group membership, instead presenting ‘stumbling across’ or attending to help others | *Need for purpose*  emotional vs. informational - men portray preference for action and purpose over emotional sharing but this may not reflect true preference;  preferences for focus and format – men portray preference for activities and distance selves from certain activities considered feminine (e.g. emotional sharing) due to implications for identity;  reciprocity and legitimising use - women happy to report motivation of seeking help whereas men prefer to legitimise as offering help to others; men deny agency in seeking help and may be influenced by other men to access support  *Becoming an expert*  limited informed choice – some men value agenda of intervention, e.g. to educate other, raise awareness, undertake lobbying and advocacy |
| Smith 2002[[99](#_ENREF_99)] | 1) men may report not needing support groups due to accessing support from existing networks (e.g. church, family); 2) men may obtain information via other sources (e.g. friends and family with experience of LTC, HCPs, literature, Internet, American Cancer Society) instead of groups; 3) men may not access due to denial of disease; 4) men may not access due to feeling uncomfortable discussing ‘sensitive issues such as sexuality with strangers’; 5) men may not access due to being ‘too busy’ with other organisations and activities | *Need for purpose*  changing needs – rather than being self-sufficient, there is less need for support if strong existing network, and possible cultural preferences exist for favouring support via church, family and friends  *Value of peers*  comparison, meaning and adjustment – some do not access intervention because do not want to identify with condition;  who is a peer – other men viewed as strangers not peers and preference for instead accessing those with experience of LTC within existing networks; do not need intimacy with others outside group |
| Sullivan 2003[[51](#_ENREF_51)] | Presents findings as consistent with 'western society's accepted forms of gendered communication' whereby men value instrumental and informational support whereas women value emotional.  Men's themes: 1) 'as we all know' (men use technical information and medical jargon, place emphasis on medical reports and knowledge, including discussion of sexual aspects); 2) 'I do not respond to messages without a PC digest' (HCPs contribute and expect men to provide case histories that conform to standards; men are expected to read key articles and be well informed); 3) 'if you want to be a partner in your own healing' (patients should 'arm' selves with up-to-date medical information to become active patients and have informed decision-making)  Women's themes: 1) 'I send my good vibes' (women have positive optimistic interactions, expressing affection); 2) 'at this cyber this party' (understanding is only possible by others with similar experiences; similar others validate and normalise experiences); 3) 'feel free to rant' (women vent feelings and frustrations about HCPs, treatment, side effects, bodily changes, relationships); 4) 'put a face with a cyber name' (women seek contact outside discussion boards and establish 'personal relationships' through remembering important treatment dates etc.); 5) 'may we all be blessed with the ability to contribute to others' lives' (women provide support to others as a form of coping, give each other advice to become active patients in their own healthcare, educate each other about condition and HCP interactions and help to 'interpret' information) | *Need for purpose*  emotional vs. informational - men highly value instrumental/informational and women highly value emotional however there is overlap between informational and emotional support; emotional support may be under-recognised in men due to happening in different ways  *Trusted environments*  group dynamics and rules of talk – men were expected to learn the rules of talk and provide case histories that conform to standards;  group facilitators – communication may be influenced by HCP presence in men’s forums  *Value of peers*  sense of community – peers validate and normalise experiences; some women view that certain understanding is only possible by others with similar experiences; some women seek contact outside discussion boards;  information, education and motivation – patients learn from each other about the condition and HCP interactions  *Becoming an expert*  health literacy and desire for information – men are expected to read key articles and be well informed; postings use technical language and jargon but this may be partly to convey power;  limited informed choice - patients should become active patients and have informed decision-making by 'arming' selves with up-to-date medical information |
| Trapp 2013[[75](#_ENREF_75)] | 1) predominant approach coping styles were ‘seeking guidance and support’ (valuing opportunity for support outside the family and the importance of shared understanding), ‘seeking information’ (focusing on learning from each other, with education happening alongside ‘connection’), and ‘acceptance’ (including dealing with uncertainty and accepting the diagnosis); 2) ‘seeking emotional support’ was the central emotion-focused coping style (this overlapped with ‘seeking guidance and support’ but was primarily concerned with emotional needs; men valued 'mutual give-and-take' ‘transactions’; 3) preferences in group qualities included an interest in connection (contrary to perception of 'solitary and emotionally restricted'), an interest in mixed sex groups (to increase discussion and to help understand perspectives of others, including family and friends), and interest in mixed diagnoses (to give 'deeper understanding' and meet practical needs of having few with shared diagnosis) | *Need for purpose*  emotional vs. informational - connection more valued than education and education can happen without connection;  reciprocity and legitimising use – men value mutuality, ‘give and take’ ‘transaction’ rather than solely receiving support  *Value of peers*  sense of community – peers offer connectedness and shared understanding; men value 'true friendships';  comparison, meaning and adjustment – group facilitates psychological adjustment and acceptance;  who is a peer - mix of diagnoses (including type and stage of cancer) may facilitate wider perspectives;  presence of women and significant others - peer support allows men to protect family and friends; mixed sex may increase discussion and perspectives and for understanding experiences of friends and family  *Becoming an expert*  who is expert - peers viewed as experts |
| Vanable 2012[[76](#_ENREF_76)] | 1) ‘focus[ing] exclusively on safer sex may not be well received’ – some men perceive negativity and blame around safer sex whereas it should be everyone’s responsibility; 2) ‘preference for a supportive, group approach that addresses other coping challenges as well as sexual risk reduction’ – appeal of informal sociable group where meet other HIV+ men, have interactive engaging group discussion, learn about each other, support each other to live ‘healthier lives’, without feeling stigmatised (by virtue of being around other HIV+ men) or ‘preached at’, instead having a facilitator but being able to ‘steer’ discussions | *Need for purpose*  preferences for focus and format – value different focus and activities with emotional sharing happening via these activities;  changing needs – social support especially important if facing isolation  *Trusted environments*  Group facilitators - facilitators are ‘needed’ (details not given)  *Value of peers*  sense of community – do not feel stigmatised when around others with same condition;  information, education and motivation - receptive to information exchange and learning from peers due to truly understanding and not feeling ‘preached at’;  who is a peer – importance of peers concerning condition and sexuality  *Becoming an expert*  health literacy and desire for information – value holistic focus rather than restriction to safe sex;  who is expert – view peers as sources of information; do not want to feel ‘preached at’ but rather that men can ‘steer’ the group |
| Wallace 2007[[69](#_ENREF_69)] | 1) some men seek support from family and friends with experience of prostate cancer; 2) HCPs are not a good source of psychosocial support – support from peers is preferred; 3) some men lack awareness of services; 4) support groups offer opportunity to meet peers going through same thing; 5) support should be available immediately after diagnosis to aid decision-making; 6) men want independent support groups due to agendas of HCPs; 7) men want to meet peers with range of treatment experiences to inform decision-making; 8) men want both 1-to-1 and peer and group support; 9) men want to see other men who are well years after treatment; 10) access may be limited by viewing ‘prostate cancer as a private matter involving male potency and urinary function’ | *Need for purpose*  changing needs – may not need intervention if strong support from family or friends; family and friends may help to access intervention; different information is needed early on for decision-making  *Value of peers*  sense of community – value peers as ‘going through same thing’;  comparison, meaning and adjustment - survivors offer self-comparison;  information, education and motivation – value ‘living examples’ and role models and would like access to men with a variety of treatment experiences to learn from;  presence of women and significant others – some men prefer support from family or friends with experience  *Becoming an expert*  who is expert – some may prefer emotional support from peers than HCPs; some men want multiple interventions and value both one-to-one and support groups, especially when decision-making;  limited informed choice – some men may be unaware of service or its nature; support groups may be viewed as neutral (provided that they are independent) unlike HCPs who may have agendas |

Notes: CAM = complementary and alternative medicine; GP = General Practitioner; HCP = healthcare professional
